# Immunoregulation in Skull Defect Repair with a Smart Hydrogel Loaded with Mesoporous Bioactive Glasses

**DOI:** 10.34133/bmr.0074

**Published:** 2024-09-06

**Authors:** Shiguo Yuan, Boyuan Zheng, Kai Zheng, Zhiheng Lai, Zihang Chen, Jing Zhao, Shaoping Li, Xiaofei Zheng, Peng Wu, Huajun Wang

**Affiliations:** ^1^ Department of Orthopaedic, Hainan Traditional Chinese Medicine Hospital, Hainan Medical University, Haikou, 571924, China.; ^2^ Department of Orthopaedic, Guangdong Provincial Hospital of Chinese Medicine, Hainan Hospital, Guangzhou University of Chinese Medicine, Guangzhou, 510388, China.; ^3^ Department of Sports Medicine, The First Affiliated Hospital, Guangdong Provincial Key Laboratory of Speed Capability, The Guangzhou Key Laboratory of Precision Orthopedics and Regenerative Medicine, Jinan University, Guangzhou, 510630, China.; ^4^ Department of Psychology, Li Ka Shing Faculty of Medicine, State Key Laboratory of Brain and Cognitive Sciences, The University of Hong Kong, Hong Kong SAR, 999077, China.; ^5^ State Key Laboratory of Quality Research in Chinese Medicine, Institute of Chinese Medical Sciences, Department of Pharmaceutical Sciences, Faculty of Health Sciences, University of Macau, Macau SAR, 519000, China.; ^6^ Department of Orthopedics, Shanghai Tenth People’s Hospital, Tongji University School of Medicine, Shanghai, 200072, China.

## Abstract

Skull defect repair is a complex and critical medical challenge, and there is an urgent need to develop multifunctional tissue engineering scaffolds for skull regeneration. The success of bone tissue engineering depends on the construction of scaffolds that can regulate the immune microenvironment of bone regeneration and mimic the liquid crystal and viscoelastic properties of natural bone extracellular matrix. Hence, a smart hydrogel (PEGDA5/AM15/CLC-BMP-4@MBG) with good biocompatibility and the ability to modulate the wound immune microenvironment has been developed for the repair of skull defects. The hydrogel consists of chitin liquid crystal hydrogel (PEGDA5/AM15/CLC) and mesoporous bioactive glasses (MBGs) loaded with bone morphogenetic protein-4 (BMP-4). The liquid crystal hydrogel not only offers the necessary biological support and mechanical properties but also maintains the stability of the liquid crystal state, facilitating adhesion and regeneration of surrounding bone tissue. In addition, BMP-4@MBG intelligently regulates the release rate of BMP-4 in response to changes in wound microenvironment, thus effectively promoting the transformation of macrophages from M1 to M2 macrophages. At the same time, Ca^2+^ and Si^4+^ released by MBG degradation and BMP-4 synergically promote bone repair process. The PEGDA5/AM15/CLC-BMP-4@MBG hydrogel shows excellent immunomodulatory and osteogenic properties of bone microenvironment and is a promising scaffold material for bone tissue engineering.

## Introduction

Skull defects are a significant clinical issue typically resulting from trauma, illness, or surgical procedures [1,2]. Traditional skull repair methods include autologous tissue transplantation and combined osteogenic material. However, these methods have some limitations, such as potential damage to the donor area, surgical complications, and incomplete repair [3,4]. In recent years, hydrogel materials, as a new biomaterial, have attracted wide attention [5–7]. Its excellent biocompatibility, bioactivity, and immunoregulatory properties make it a cutting-edge material in the field of skull defect repair [8,9].

The natural bone extracellular matrix (ECM) environment is an important part of bone tissue, which is a 3-dimensional (3D) network structure composed of a series of complex molecules [10,11]. ECM environment provides structural support and bioactive microenvironment for bone cells and regulates the physiological function and cell behavior of bone cells [7,12]. Modeling and understanding the ECM environment is important for developing biomaterials for bone tissue repair and regeneration. The majority of studies on bone repair materials conducted recently have been on the creation of different composite materials containing inorganic biomineralizing elements that resemble bone [13–15], such as the biomineralizing modification of natural or synthetic materials such as collagen [16,17], polysaccharide [18], and polylactic acid [19]. However, the majority of reported composite materials used for repairing bone defects lack the liquid crystal state found in the ECM of bone. The main organic component of bone ECM, type I collagen (COL-I), is an insoluble fibrin with unique liquid crystal properties achieved through self-assembly [20]. As a result, these composites are incapable of reproducing the anisotropic attributes found in natural bone ECM [21]. At the same time, they also cannot play the ability to promote bone formation. Collagen exists as an anisotropic liquid-crystal state. Collagen controls the deposition of inorganic mineral salts in addition to directing osteoblastic cell adhesion, migration, proliferation, and differentiation. Collagen also helps to build bones and facilitate mineralization, contributing to the development of a structured multilevel architecture, thus providing bone tissue with exceptional mechanical strength and biological functionality [22,23]. Price et al. drew inspiration from this concept and successfully developed a collagen membrane exhibiting a cholesteric liquid crystal state utilizing collagen protein. This innovative approach effectively promotes osteogenic differentiation and guides the directed growth of cells [24]. Although a collagen membrane with a cholesteric liquid crystal state has been successfully developed, its restricted forming ability and insufficient mechanical strength severely limit its practical applicability in bone tissue healing.

**Fig. 1.**
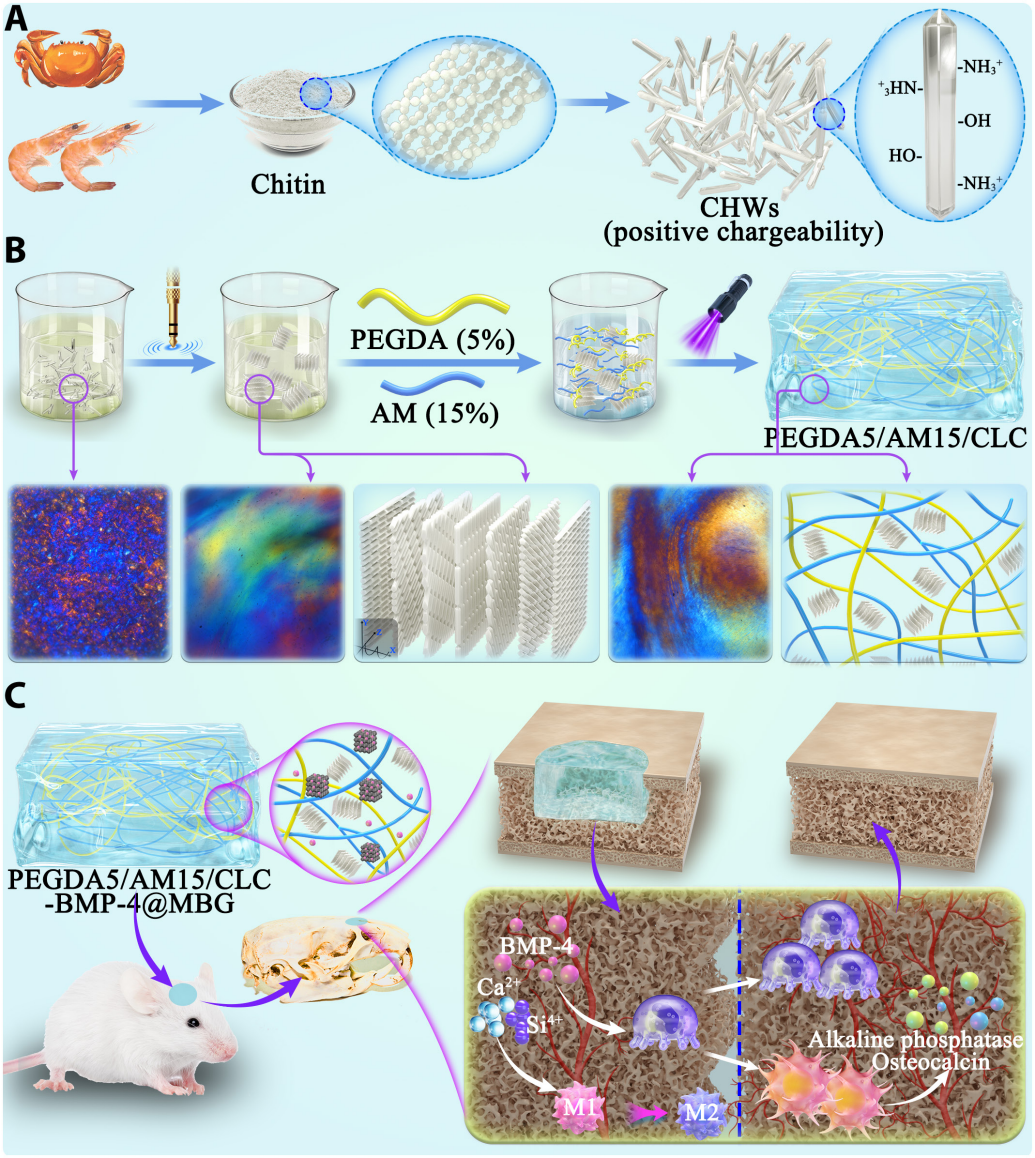
Preparation process and mechanism of liquid crystal hydrogel. (A) Schematic diagram of preparation of CHWs and (B) liquid crystal hydrogel. (C) Schematic illustration of liquid crystal hydrogel promoting skull repair.

Chitin is a natural polymer derived from seafood such as shrimp, crab, lobster, and other crustaceans. Due to its special chemical structure and biological activity, chitin has been widely concerned and studied, has been applied in many fields such as stomatology and orthopedics, and has achieved certain results [25,26]. Chitin whiskers (CHWs), which are typically obtained through the hydrolysis of chitin in a strong acid or alkali solution, possess outstanding cell affinity, antibacterial, and osteogenic properties (Fig. 1A). Moreover, under certain conditions, they can self-assemble to form a liquid crystal state with similarities to the natural bone ECM [27–29]. However, the morphology of CHW liquid crystal suspension is unstable, which hinders its further application in the field of bone repair. Moreover, a single component of chitin liquid crystal is difficult to reverse the inflammatory reaction caused by bone tissue injury. Mesoporous bioactive glass (MBG) exhibits excellent biocompatibility and bioactivity, facilitating the adhesion and proliferation of osteoblasts while supporting the formation of new bone [15,30]. This porous material not only accommodates a range of pharmaceuticals and growth factors but also provides ample space and support for osteoblast growth, thereby accelerating the healing of bone defects and being highly sought after in bone regeneration [31,32]. In addition, due to their acid-responsive degradation, MBG can effectively modulate the controlled release of encapsulated drugs [33]. Moreover, the Ca^2+^ and Si^4+^ from its self-degradation interact synergistically to regulate the immune microenvironment within bone tissue (facilitate the regeneration and repair of bone matrix), thereby facilitating bone repair processes [34].

Therefore, a smart liquid crystal hydrogel loaded with MBG and bone morphogenetic protein-4 (BMP-4) with good biocompatibility and immunomodulatory activity was designed by combining viscoelastic hydrogel with liquid crystal CHW suspension in this paper. When exposed to ultraviolet (UV) irradiation, polyethylene glycol diacrylate (PEGDA) and acrylamide (AM) rapidly form hydrogels with the aid of photoinitiators, with CHW liquid crystal getting frozen in the hydrogel network (Fig. 1B). This liquid crystal hydrogel not only offers appropriate biological microenvironment and mechanical properties support but also maintains stability of CHW liquid crystal state and promotes adhesion and regeneration of surrounding bone tissue. More specifically, MBG adjusts its own degradation rate in response to changes in the bone microenvironment and intelligently regulates the release of BMP-4 attached to the surface of MBG, thus promoting the polarization of M2 macrophages. At the same time, Ca^2+^ and Si^4+^ released by MBG degradation and BMP-4 synergistic effect further promote bone repair process (Fig. 1C). This liquid crystal smart hydrogel holds immense potential for its utilization in the field of bone tissue regeneration.

## Materials and Methods

### Materials

Chitin was sourced from Shanghai Aladdin Chemical Co., Ltd. AM (AR) and PEGDA (*M*w = 800) were purchased from Macklin Biochemical Technology Co. Ltd. Lithiumphenyl-2, 4, 6-trimethyl-benzoylphosphinate (LAP; 97%) was provided by Shanghai Bide Medical Technology Co. Ltd. Tetraethoxysilane (TEOS; 95%), poly(ethylene-propylene oxide) trioctyl silane (P123, AR), triethyl phosphorus triacetate (TEP, AR), and hydrochloric acid (HCl, AR) were purchased at Guangzhou Chemical Reagent Factory. BMP-4 was purchased from Sigma-Aldrich. Every other material and solvent utilized was of the quality of an analytical reagent.

### Preparation of CHWs

In the initial step, a 3.0 mol/l solution of hydrochloric acid was mixed with 20 g of chitin powder. This mixture was subjected to a 3-h reaction in an oil bath that was kept at 105 °C. After the reaction was finished, the solution was allowed to cool to ambient temperature before being centrifuged for 10 min at 4,000 rpm. The resulting supernatant was carefully poured off, leaving behind the desired materials. The crude product of CHWs was transferred to a dialysis bag and dialyzed for a week. After undergoing the freeze-drying process, pure CHWs were obtained and subsequently stored in a drying cabinet for future utilization and preservation. Next, the prepared CHWs were dispersed in anhydrous ethanol and subjected to 30 min of ultrasonication. The resulting suspended droplets of CHWs were placed onto a copper mesh, and after air drying, the morphology of the CHWs was examined using a transmission electron microscope (TEM, Stemi 2000-C, Germany). In addition, the size distribution of CHWs was determined by measuring the size of 200 randomly selected CHWs through TEM images using ImageJ software. Meanwhile, the crystalline structures of chitin and CHWs were analyzed through the use of x-ray diffractometry (XRD, Mini Flex 600, Japan). Also, the zeta potential values of 0.05 wt% CHW aqueous suspension were examined at various pH levels using a zeta potential analyzer.

### Synthesis and characterization of MBG and BMP-4@MBG

MBG was synthesized following previously outlined protocols [35]. In brief, a solution comprising 60 ml of ethanol, TEOS (6.7 g), P123 (4.0 g), TEP (0.36 g), 0.5 M HCl (1.0 g), and Ca(NO_3_)_2_·4H_2_O (1.4 g) was stirred at 25 °C for 24 h. The resulting sol underwent evaporation-induced self-assembly and was subsequently vacuum-dried for 24 h. Removal of the P123 structure-directing template involved calcination at 600 °C in air for 6 h, with the temperature ramped up at a rate of 1 °C/min. Morphological characterization of the MBG samples was conducted using a scanning electron microscope (SEM; SU9000, Hitachi, Japan) and TEM. The MBG was dispersed into a phosphate-buffered saline (PBS) solution (1 mg/ml), followed by the addition of BMP-4 (10 μg/ml), and then stirred at 4 °C overnight, centrifuged, and filtered, and the obtained filter residue (BMP-4@MBG) was freeze-dried and stored in the refrigerator at −20 °C.

### Preparation of chitin liquid crystal and hydrogels

The CHW (1 g) was added to 10 ml of deionized water, and the chitin liquid crystal was obtained after 150 min (450 W) ultrasound with a cell crusher. Next, 1.8 ml of the prepared chitin liquid crystal was taken and mixed with 0.1 g of PEGDA and 0.3 g of AM until fully dissolved. Then, 200 μl of LAP solution (2.5%) and BMP-4@MBG were added to the mixture to obtain the PEGDA5/AM15/CLC-BMP-4@MBG precursor solution. After the precursor solution was added into a mold, it was exposed to UV light from a lamp for 10 min. As a result, the PEGDA5/AM15/CLC-BMP-4@MBG hydrogel was formed. Hydrogels containing other components were prepared using a similar process. Specifically, the PEGDA5/AM15/CHW precursor liquid or hydrogel was prepared without ultrasound by the cell fragmentation in the preparation process and without the presence of liquid crystal. The liquid crystal phenomenon of the material was observed by a polarizing microscope (BX53M, Japan).

### Mechanical tests

Tensile and compression tests were performed on the hydrogels using a Shimadzu universal testing instrument. The hydrogel was initially molded into the shape of a dumbbell, measuring 30 mm in length, 4 mm in width, and 2 mm in thickness, in preparation for the tensile test. The sample was then evaluated for tensile strength at a rate of 10 mm/min while being stretched in a fixture. By measuring the elongation and stress at the fracture point, respectively, the hydrogel’s breaking strain and tensile strength were ascertained. The hydrogels were shaped into cylinders with a diameter of 10 mm and a height of 10 mm for the compression test. The compression test was then conducted at a rate of 1 mm/min. By measuring the compression ratio and the stress at the fracture point, respectively, the hydrogel’s breaking strain and compressive strength were determined. Additionally, 30 cycles of compression at a rate of 4 mm/min were performed using the PEGDA5/AM15/CLC hydrogel.

### Microscopic morphology observation

After the hydrogel samples were prepared, they were prefrozen at −80 °C for 2 h to allow the water in the internal structure to form ice crystals. The samples were then placed in a vacuum freeze dryer and frozen for 48 h, enabling the ice crystals to sublime. The SEM was utilized to capture the internal morphology of the resulting freeze-dried hydrogels.

### Rheological testing

The rheological properties of both hydrogels and their precursors were analyzed using a rotating rheometer. The precursor solution was subjected to an angular frequency of 10.0 rad/s and a constant strain rate of 1.0% throughout time. Furthermore, frequency sweep oscillation experiments covering an angular frequency range of 0.1 to 100 rad/s were carried out, maintaining a constant strain of 1%. Oscillating amplitude sweep tests were also performed while maintaining 1-Hz frequency, with strain levels ranging from 0.001% to 10%.

### Swelling measurement

Immersing the hydrogel in PBS at 37 °C and a pH of 7.4 allowed researchers to examine its swelling behavior. The hydrogel sample’s initial weight, *W*d, was determined prior to the swelling test. After carefully wiping away any extra water from the surfaces using a filter paper at predetermined intervals, the swollen sample was then weighed as *W*s. Eq. 1 was used to compute the swelling ratio (SR).

$$\mathrm{SR}=\frac{Ws-Wd}{Ws}\times 100\%$$ (1)

### Degradation and release experiments in vitro

The release performance of small-molecule medications in hydrogels was investigated using rhodamine B (Rh B) as a model drug. Initially, the absorbance at 540-nm wavelength was measured using a UV-visible spectrophotometer. This measurement was taken after preparing different concentrations of Rh B aqueous solution. Subsequently, a standard curve was constructed using the absorbance values of Rh B in the aqueous solution along with their corresponding concentrations. Rh B (0.1%) was incorporated into the hydrogel precursor solution. Subsequently, 0.8 ml of the precursor solution were injected into the mold, and the hydrogel was formed through exposure to UV light. Each individual hydrogel was then submerged in 15 ml of PBS solution. The hydrogel solution (3 ml) was taken out of the system at prearranged intervals, and its place was filled with an equivalent volume of brand-new PBS solution. This process was repeated to collect samples at different time points, allowing for the measurement of the cumulative release of Rh B over time. By plotting the measured values, a release curve was constructed to visualize the release behavior of Rh B from the hydrogel.

Then, the degradation of MBG (Ca^2+^ release) in different pH solution environments (pH 4.0, pH 7.4, and pH 9.0) was investigated. To put it simply, first, 50 mg of MBG was weighed into a 15-ml centrifuge tube; then 10 ml of solution of different pH was added, mixed well, and put into oven at 37 °C. At 1, 2, 4, 6, and 8 h, 200 μl of supernatant was centrifuged, and 200 μl of solution was added at the same time. The quantitative supernatant was mixed with 1% sodium oxalate solution, and the degradation of MBG was initially determined according to the turbidity of the solution. Then, the 96-well plate was added with 10 μl of acid chromium blue k solution, 170 μl of sodium hydroxide solution (pH 10), and 20 μl of supernatant. Optical density (OD) values were measured at 450 nm after reaction for 5 min. Finally, Ca^2+^ release was calculated according to the standard curve. In addition, the degradation of MBG samples in PBS solution for a long time (20 d) was also investigated. In short, a 50-mg sample of MBG was placed in a 10-ml PBS solution, and the content of Ca^2+^ and Si^4+^ in the supernatant was measured at regular intervals using inductively coupled plasma mass spectroscopy (Agilent 5800, USA).

### Cytocompatibility test

The mouse preosteoblast cell line (MC3T3-E1) was used to assess the hydrogel system’s biocompatibility. The hydrogel scaffolds were sterilized for cell culture by being submerged in 75% alcohol for 30 min and exposed to UV radiation for 2 h. A mouse preosteoblast cell line (MC3T3-E1) was employed to evaluate the biocompatibility of the hydrogel system. The hydrogel scaffolds were treated to UV light for 2 h and immersed in 75% alcohol for 30 min in order to sanitize them for cell culture. In the cell culture experiments, 1 × 10^4^ cells per well of the hydrogel samples were cocultured with MC3T3-E1 cells. Cell Counting Kit-8 tests were used to measure the cell proliferation on the hydrogels after 1, 3, and 5 d of culture. Using a microplate reader, the OD values were determined at 450 nm. Live/dead cell staining was used to visualize the viability of the cells, and inverted fluorescence microscopy (Olympus CKX41, Japan) was used to view the cell state. It was possible to distinguish between living and dead cells because to this staining. Additionally, using confocal laser scanning microscopy (CLSM, Zeiss-LSM 510, Germany), the morphology, cytoskeletal organization, and spreading of the MC3T3-E1 cells cocultured with the hydrogel samples were examined. With the use of this imaging method, specific details regarding the cell shape and interactions with the hydrogel scaffolds could be obtained.

### Cell migration experiment

To evaluate cell migration, the MC3T3-E1 cells were cultured in 24-well plates at a density of 3 × 10^5^ cells per well for 1 d. Subsequently, utilizing a 1-ml sterile plastic pipette, we conducted a scratch assay and introduced various components and serum-free culture medium into the wells for cocultivation over a 48-h duration. The cells were stained with calcein-AM / propyl iodide (keyGEN, China), and their migration was tracked using an inverted fluorescence microscope. We calculated the cell mobility ratio (MR) using Eq. 2.

$$\mathrm{MR}=\frac{L_{0}-L_{t}}{L_{0}}\times 100\%$$ (2)

*L*_0_ refers to the initial width of the cell scratch, while *L_t_* represents the width of the scratch after the cells have been cultured for *t* h.

### Detection of osteogenic gene expression

By measuring the alkaline phosphatase (ALP) activity of cells cultivated on the hydrogels for 14 d using an ALP kit and a bicinchoninic acid (BCA) protein assay, the impact of hydrogels on MC3T3-E1 cell differentiation was examined. Mineralization was evaluated by measuring the amount of calcium phosphate produced by cells grown on the hydrogels for 14 and 21 d using an Alizarin red staining kit. Moreover, the effect of hydrogel on the expression of genes associated to osteogenesis in MC3T3-E1 cells was evaluated using real-time quantitative polymerase chain reaction (RT-qPCR). Samples of MC3T3-E1 cells cultivated on the materials after 7 and 14 d were used to evaluate the expression of Runt-related transcription factor 2 (Runx-2), osteopontin (OPN), COL-I, osteocalcin (OCN), and other components. An internal control was provided by the glyceraldehyde-β-actin gene. The primer sequences for the genes can be found in Table S1 (Supplementary Materials). Each experiment was conducted 3 times, and data analysis utilized the 2^-ΔΔct^ method.

### In vitro assessments of macrophage polarization

After being injected onto cell culture plates, RAW264.7 cells were subjected to 3 different treatments for 48 h: control, PEGDA5/AM15/CLC, and PEGDA5/AM15/CLC-BMP-4@MBG. The cells were separated by centrifugation, and then they were stained using anti-CD206, anti-F4/80, and anti-fluorescein isothiocyanate antibodies, as well as 2 rounds of cold PBS washing. The percentage of polarized macrophages was ascertained by flow cytometry (C6, BD, USA).

About the RT-qPCR investigation, 2.0 × 10^5^ RAW 264.7 was seeded into the 6-well plate, and the cells were treated for 24 h with 5 ng/ml of interleukin-1β (IL-1β). RT-qPCR was utilized to assess the cytokine levels for M1 macrophages (tumor necrosis factor-α [TNF-α]) and M2 phenotypes (transforming growth factor-β [TGF-β] and IL-10) after treatment with various formulations.

### In vivo animal study

Our animal procedures were conducted following the Guidelines for the National Research Council’s Guide for the Care and Use of Laboratory Animals and were approved by the Animal Ethics Committee of Jinan University. Thirty-six male, healthy, 200- to 250-g Sprague Dawley rats were split into 3 groups at random, with 4 Sprague Dawley rats in each group. In the blank control group, only the skull deformity was treated with saline. Material control groups, namely, PEGDA5/AM15/CLC and PEGDA5/AM15/CLC-BMP-4@MBG, were implanted after the surgery to repair a skull defect. A 5-mm circular bone hole was drilled into the bone in the cranial area, and a hydrogel material was then filled into the bone defect. The recovery of the defect site was monitored at 3 different time points after the surgery: 4, 8, and 12 weeks. Using a micro-computed tomography (CT) scanner (Skyscan 1176, Kontich, Belgium), the 3D structures of the regenerated bone tissue inside the skull defect area were carefully examined. Subsequently, the compiled dataset was skillfully reconstructed into visually stunning and accurate 3D images with the aid of the CTVox program, developed by the Skyscan Company.

### Histological observation

Following the collection of a tissue sample from the skull defect, it was placed in a 4% paraformaldehyde solution and refrigerated at 4 °C for 72 h. The sample was then placed in a bone tissue decalcification solution for approximately 45 d, with the decalcification solution being changed weekly. Subsequently, the sample was immersed in PBS for 2 h and underwent dehydration using a gradient ethanol solution for 24 h. The sample was then embedded in paraffin after adding xylene and sectioned into 5-μm-thick slices along the longitudinal section of the skull. The slices of skull defect tissue that were obtained were subjected to hematoxylin and eosin (H&E) staining as well as Masson’s trichrome staining techniques. These staining techniques are commonly used to evaluate tissue structures, collagen content, and cellular morphology. The animal’s primary organs, such as the liver, kidneys, lungs, spleen, and heart, were then removed and imbedded in paraffin. After the sectioning and H&E staining process, the embedded organs were prepared for further histological examination.

### Immunohistochemistry and immunofluorescence analysis

The tissue sections obtained from the sample of skull defects underwent immunohistochemical examination in addition to histological staining. This process involved the localization and visualization of specific proteins in the tissue using targeted antibodies. Specifically, antibodies against Runx-2 and OCN were utilized for immunohistochemistry on the tissue sections, while CD86, CD206, CD31, and α-smooth muscle actin (α-SMA) were employed for immunofluorescence labeling. The staining procedures followed each antibody’s specific standard technique. These techniques played a crucial role in elucidating the cellular and molecular alterations that take place in the tissue surrounding the skull defect.

### Statistical analysis

In the statistical study, 1-way analysis of variance was employed to compare across multiple groups, while 2-way *t* tests were used for comparisons between 2 groups. The level of significance was set at *P* < 0.05 (*), *P* < 0.01 (**), and *P* < 0.001 (***).

## Results

### Preparation and characterization of CHWs

**Fig. 2.**
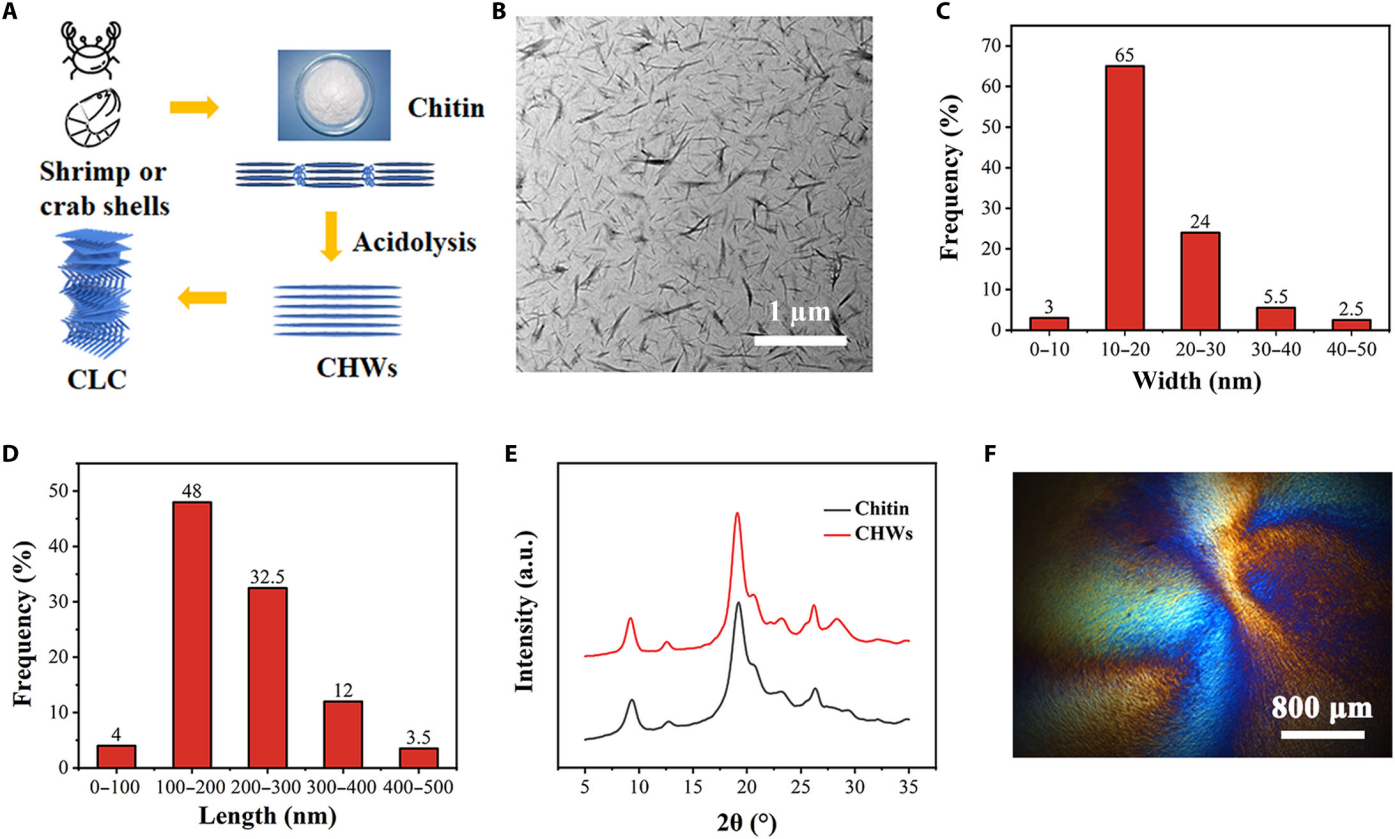
Preparation and characterization of CHWs. (A) Schematic illustration of CHWs. (B) The TEM image of CHWs. (C and D) Statistical results of length and width distribution of CHWs. (E) XRD image of chitin and CHWs. (F) Polarizing photo of CHW suspension.

As shown in Fig. 2A, large amounts of chitin can be immediately recovered from seafood debris, such as the shells of crabs and shrimp, and an alkali-or acid-deacetylation reaction can provide fiber CHWs [36]. CHWs can be assembled to construct a liquid crystal structure of CHWs (CLC) under external intervention [37]. The needle-like morphology of CHWs with a large aspect ratio could be detected under TEM, as illustrated in Fig. 2B. The length and width of CHWs were concentrated in 100 to 300 nm and 10 to 30 nm, respectively, based on the evaluation of the ImageJ software (Fig. 2C and D). The CHW curve showed clear distinctive diffraction peaks through XRD analysis (Fig. 2E) at 9.2°, 12.7°, 19.2°, 20.7°, 23.3°, and 26.3°, which correspond to crystal faces (020), (021), (110), (120), (130), and (013), respectively. The diffraction peaks’ position aligned with that of chitin, suggesting that α-chitin’s structure remained intact even after hydrolysis by acid [38].

**Fig. 3.**
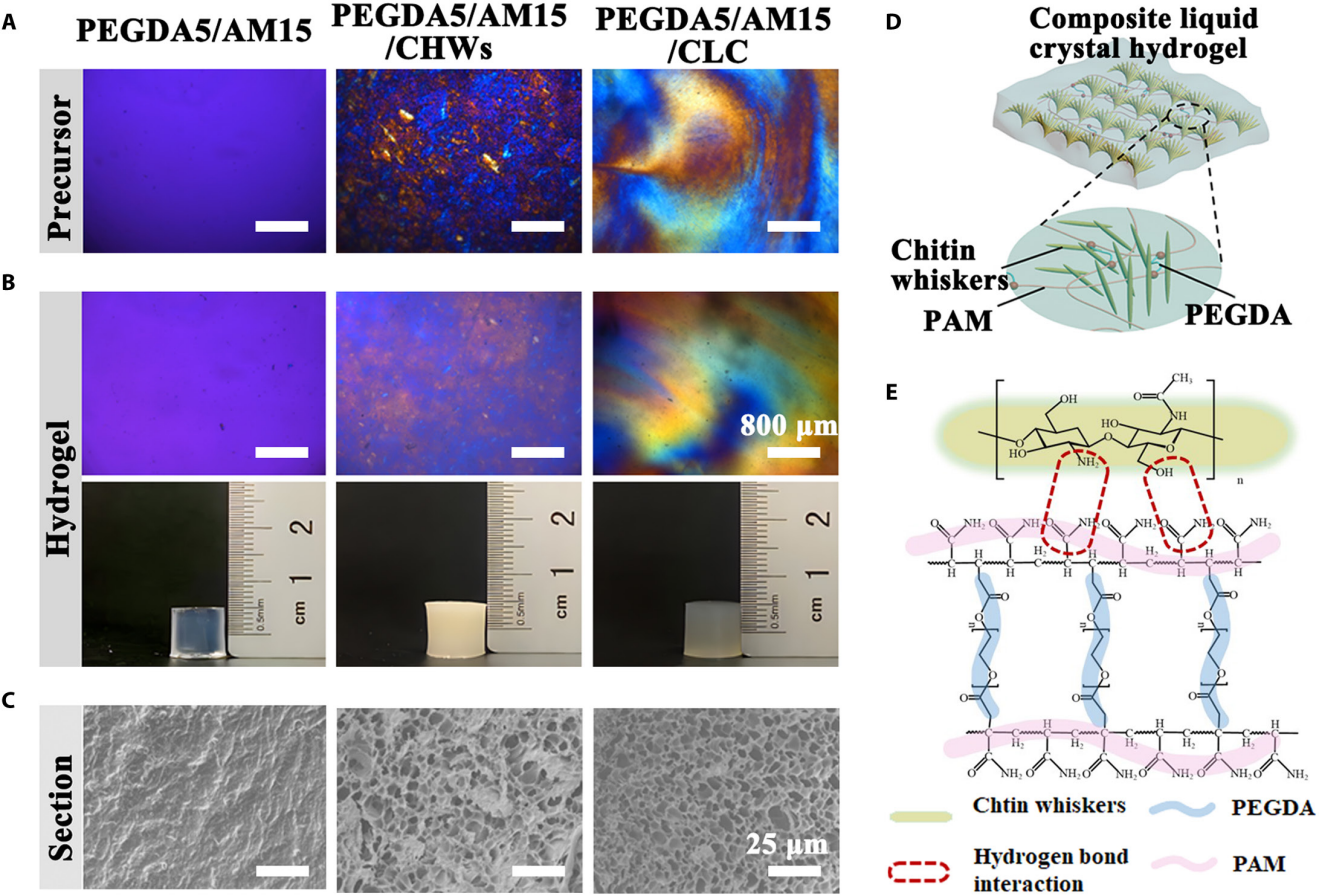
Physical properties and molecular interactions of liquid crystal hydrogels. Polarizing images of (A) precursor solutions and (B) hydrogels. (C) SEM images of hydrogel sections. (D) The structure diagram and (E) the schematic diagram of molecular interactions in a hydrogel of the PEGDA5/AM15/CLC hydrogel.

At the same time, the peak became sharper, indicating that the amorphous portion of the chitin was broken down by acid hydrolysis, leading to an increase in the crystallinity of the chitin. After 150 min of ultrasonication of 10% CHW water dispersion using a cell fragmentation apparatus, polarized microscopy revealed the fingerprint structure and colorful liquid crystal phenomena, indicating that the chitin liquid crystal was cholesteric liquid crystal (Fig. 2F).

### Liquid crystal properties of hydrogels

Due to its drawback of liquid flow and susceptibility to external influences, whisker liquid crystal suspension cannot be employed as a stand-alone material. In this work, a stable liquid crystal hydrogel was created by adding AM, PEGDA, and LAP as the second component to whisker liquid crystal solution and cross-linking it with UV light.

Subsequently, we used a polarizing microscope to characterize the liquid crystal properties of the hydrogel and its precursor. Figure 3A and B depicts polarizing images. The polarizing images of the PEGDA5/AM15/CLC precursor clearly displayed birefringence, indicating that the precursor maintained the liquid crystal state formed through whisker self-assembly. In contrast, PEGDA5/AM15/CHW precursor solution was prepared only with stirring treatment and did not exhibit the birefringence phenomenon under the polarizing microscope, indicating that the CHWs in the precursor solution did not form liquid crystal structure. Similarly, the precursors composed only of PEGDA and AM showed no birefringence at all and showed isotropy. After exposure to UV light, all the precursors underwent rapid cross-linking to form stable hydrogels. The birefringence of the hydrogels was nearly identical to that of the corresponding precursors. This suggests that the cross-linking network of PEGDA/AM had minimal impact on the crystal texture formed through whisker self-assembly, allowing the whisker to maintain its liquid crystal state with morphological stability. The internal structure diagram of the cross-linked hydrogel is depicted in Fig. 3D. It illustrates that the PEGDA cross-linking network is intertwined within the gaps of the whiskers and layers of the spiral structure, without significantly impairing the integrity of the whiskers’ spiral arrangement. The long-chain molecule polyacrylamide (PAM) in the hydrogel can also hydrogen bond with CHWs, which further enhances the stability of the liquid crystal hydrogel (Fig. 3E). Additionally, we examined the cross-sectional structure of the hydrogels. SEM images revealed that the PEGDA5/AM15/CHWs and PEGDA5/AM15 hydrogels exhibited a higher degree of regularity and a greater abundance of void structures (Fig. 3C), which is conducive to cell proliferation and differentiation and nutrient transport.

### Mechanical and rheological properties of hydrogels

**Fig. 4.**
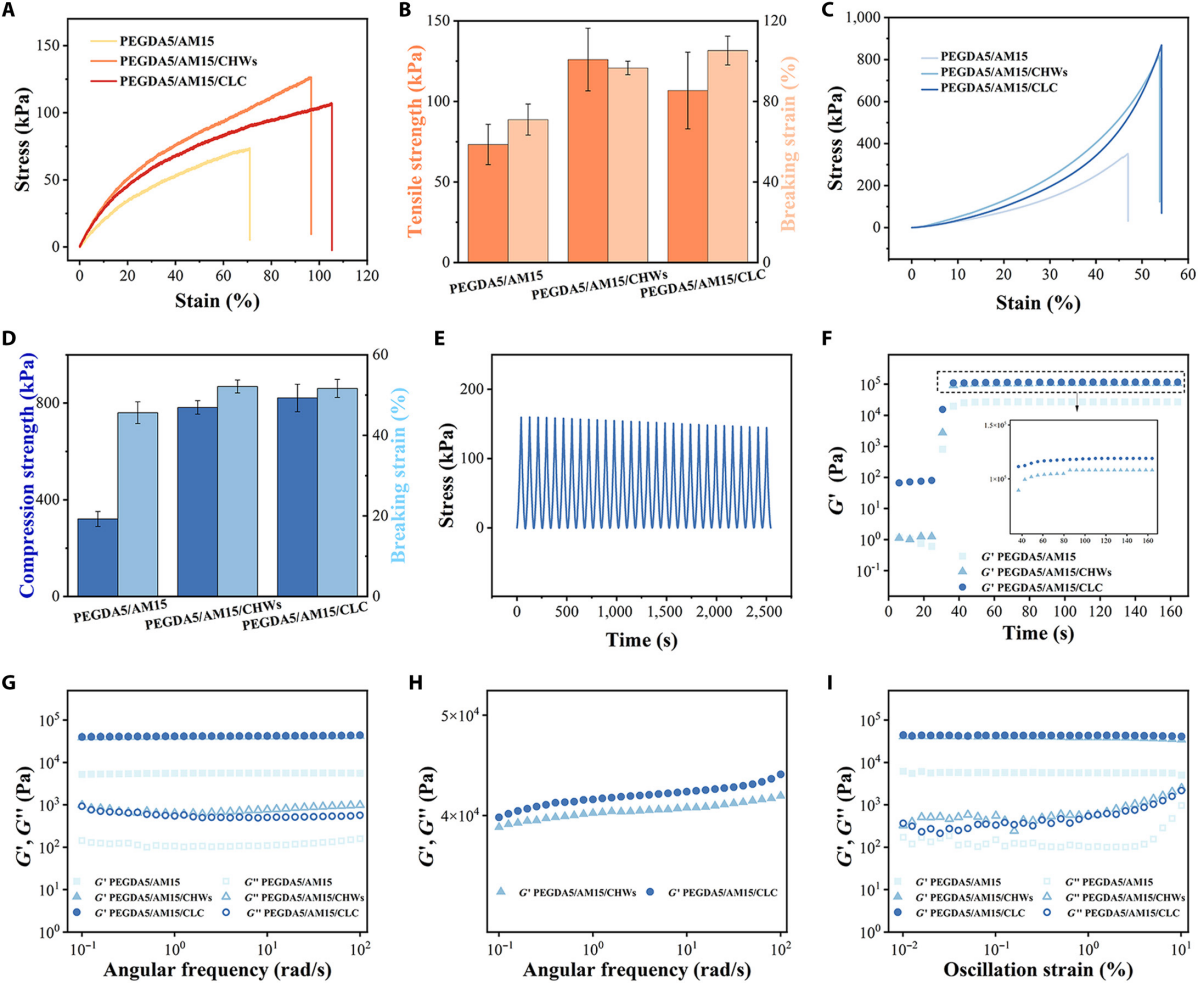
Characterization of mechanical and rheological properties of hydrogels. (A and B) Tensile test results and (C and D) of hydrogels of different components. (E) Compression cycle test result of PEGDA5/AM15/CLC hydrogel. Rheological tests: (F) Time sweep tests. (G and H) Frequency sweep tests. (I) Strain sweep tests.

To investigate the impact of whisker addition on hydrogel mechanical properties, the tensile and compression properties of hydrogels were evaluated. Compared with PEGDA5/AM15 hydrogels, the tensile strength, tensile strain, compressive strength and compressive strain of the hydrogels with whiskers were significantly increased (Fig. 4A to D). Among them, the most significant is that the tensile strain of the hydrogel increased from 70% to more than 100%, and the compressive strength of the hydrogel increased from 500 to 800 kPa. This is because the hydrogel hydrogen bond with CHWs and the electrostatic interaction between CHWs enhance the mechanical strength of the hydrogel. At the same time, due to the reversibility of the physical action, the flexibility of the hydrogel is improved, so that the hydrogel has good toughness. In addition, the directional arrangement and distribution of whiskers in the liquid crystal hydrogel are uniform, and the PEGDA5/AM15/CLC hydrogel has good mechanical stability. PEGDA5/AM15/CLC hydrogel maintained stable compression strength after 30 times of cyclic compression test (Fig. 4E). The mechanical characteristics of liquid crystal hydrogels with varying PEGDA and AM concentrations were also examined. After the liquid crystal of chitin formed hydrogel with different contents of PEGDA and AM, the gorgeous birefringence phenomenon could still be observed under polarized light, and the liquid crystal shape could still be maintained (Fig. S1). The mechanical properties of 4 different components of liquid crystal hydrogels were investigated by tensile and compression experiments. With the increase of the proportion of AM, the tensile and compressive fracture strain of hydrogel increased, showing better flexibility (Fig. S2). The average tensile strain and compressive strain of PEGDA5/AM15/CLC liquid crystal hydrogels were 105% and 52%, respectively, while the tensile strain and compressive strain of PEGDA20/AM0/CLC, PEGDA15/AM5/CLC, and PEGDA10/AM10/CLC liquid crystal hydrogels were less than 50%. In addition, the tensile strength and compressive strength of PEGDA5/AM15/CLC hydrogel reach 110 and 800 kPa respectively, which had strong rupture resistance. PEGDA5/AM15/CLC liquid crystal hydrogels, possessing both viscoelastic and liquid crystal phase, may serve as a microenvironment similar to the ECM of bone. Consequently, they are anticipated to function as exceptional bone repair materials.

Then, the rheological properties of hydrogel and its precursor were determined by a rotating rheometer. As shown in Fig. 4F, in the time scan test of rheology, the hydrogel precursor began to photocrosslink at the 30 s, and the *G*′ of all the precursor fluids rose rapidly under UV irradiation. After about 10 s, the *G*′ of the precursor liquid gradually stabilized, indicating that the hydrogel cross-link was completed at this time. At the same time, it is further demonstrated that the introduction of CHW has no obvious effect on the photocuring process of hydrogel precursor. The sample’s storage modulus (*G*′) and loss modulus (*G*″) were ascertained through measurement within the 0.1 to 100 rad/s angular frequency range. The strain applied during the measurements was kept constant at 1%. The modulus of PEGDA5/AM15/CLC and PEGDA5/AM15/CHWs was significantly higher than that of PEGDA5/AM15 (Fig. 4G). This is attributed to the enhanced mechanical strength of the hydrogel, resulting from the physical interaction between the added whiskers and hydrogel molecules. Among them, the modulus of PEGDA5/AM15/CLC was slightly higher than that of PEGDA5/AM15/CHWs (Fig. 4H). This phenomenon is attributed to the structured spiral alignment of whiskers following ultrasonic treatment, driven by electrostatic repulsion and van der Waals forces. This alignment decreases the interwhisker free volume and impedes their mutual sliding. Consequently, the *G*′ and viscosity of the precursor fluid after ultrasonic treatment are increased. The strain scan test results in Fig. 4I show that all hydrogel samples were linear viscoelastic regions in the strain range of 0.01 to 1%, which indicates that all hydrogels have good ductility.

### Swelling, degradation, and release properties of hydrogels

**Fig. 5.**
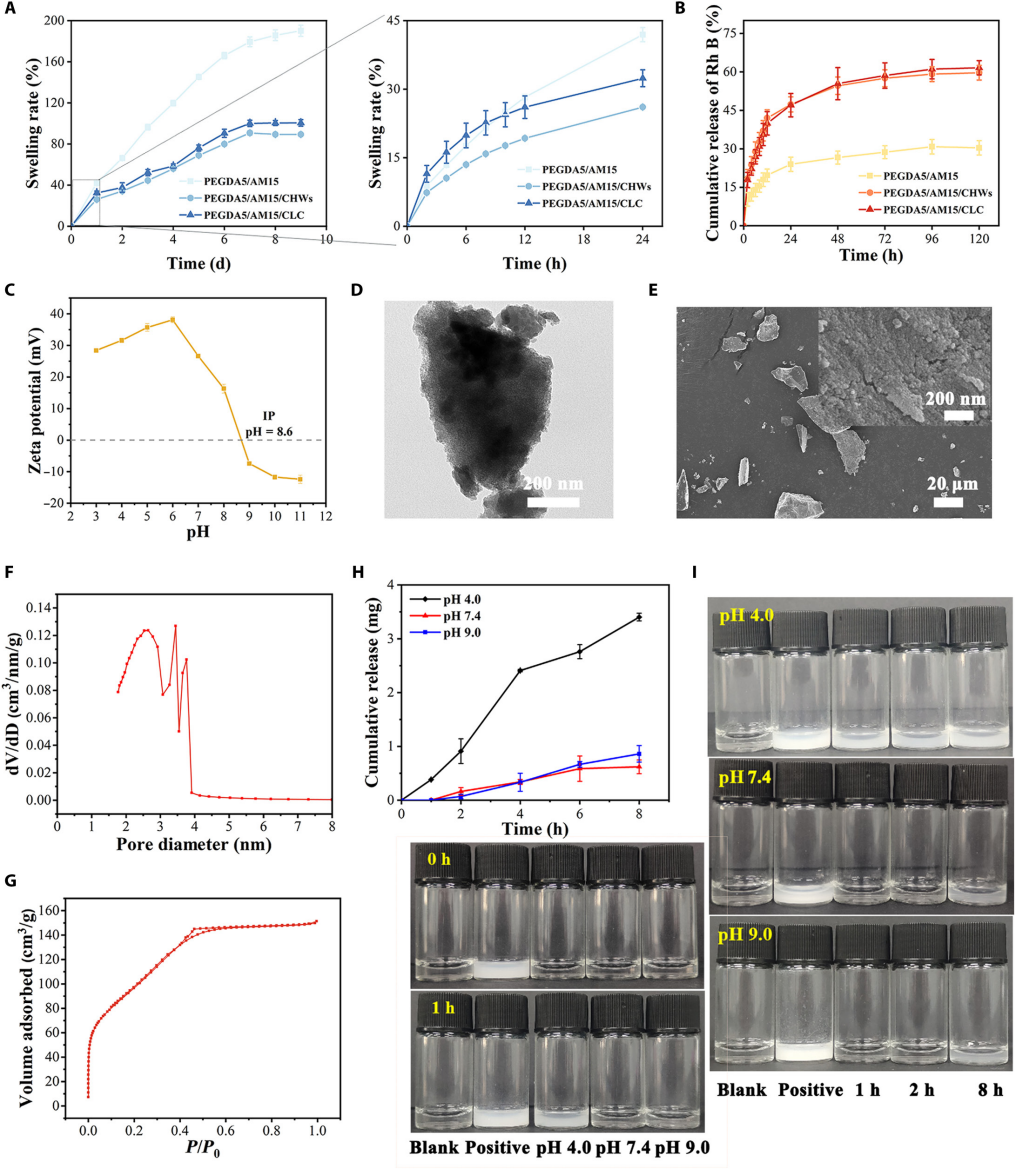
Swelling and release properties of hydrogels and characterization of MBG. (A) Swelling properties of hydrogels. (B) Cumulative release rate of Rh B. (C) Electrical properties of chitin in different pH environments. (D) TEM and (E) SEM images of the MBG. (F) Mesopore pore size distributions and (G) nitrogen adsorption–desorption isotherms of the MBG. (H) Ca^2+^ release rate and (I) degradation pictures of the MBG in different pH solution environments.

In the process of skull repair, hydrogel is used as a filling material, and its swelling performance is directly related to its stability and plasticity at the repair site. As depicted in Fig. 5A, the 3 hydrogels exhibited a slow SR, requiring 1 week to reach swelling equilibrium. Upon reaching swelling equilibrium, the SRs for PEGDA5/AM15, PEGDA5/AM15/CHWs, and PEGDA5/AM15/CLC hydrogels were 190%, 89%, and 100%, respectively. The presence of whiskers in the PEGDA5/AM15/CHWs and PEGDA5/AM15/CLC hydrogels restricts their swelling to a large extent due to interactions with PAM molecules. Notably, the whiskers in PEGDA5/AM15/CLC liquid crystal hydrogels are arranged in a regular pattern, enhancing the pore structure within the hydrogels. Consequently, the swelling property of PEGDA5/AM15/CLC hydrogels was slightly higher compared to that of PEGDA5/AM15/CHW hydrogels. Moreover, with the introduction of CHW, the degradation rate of hydrogel was increased. On the 3rd day of the hydrogel degradation test, the hydrogel still showed a dense pore structure, while on the 14th day, the pores became significantly larger and partially collapsed (Fig. S3A). PEGDA5/AM15/CLC hydrogel was immersed in dissolved enzyme solution for 21 d, and the degradation ratio reached 19.78 ± 1.49% (Fig. S3B). The proper degradation performance of scaffold materials helps cells to differentiate in situ osteogenesis and better promote bone tissue repair.

The releasing impact of Rh B in hydrogels was then examined using Rh B as a simulated drug. Rh B implanted in hydrogel at different durations was measured cumulatively by using the standard curve of Rh B aqueous solution (Fig. S4). Figure 5B illustrates that Rh B in PEGDA5/AM15, PEGDA5/AM15/CHWs, and PEGDA5/AM15/CLC hydrogels reached a state of release equilibrium after 120 h, with cumulative release rates of 30%, 59%, and 61%, respectively. PEGDA5/AM15 hydrogels, characterized by a highly dense pore structure, showed limited release of the loaded Rh B. In contrast, PEGDA5/AM15/CHWs and PEGDA5/AM15/CLC hydrogels exhibited more suitable pore structures, facilitating drug loading, release, as well as the delivery of cells and nutrients. Moreover, chitin demonstrates distinct electrical properties in various pH environments (Fig. 5C). PEGDA5/AM15/CLC hydrogels loaded with cationic drugs can intelligently control drug release as the physiological environment of the wound changes. If there is an inflammatory reaction, the environmental pH decreases, and the positive charge of the chitin band in the hydrogel becomes stronger, thus enhancing the repulsive force and accelerating the drug release. In addition, the adsorption of BCA protein by hydrogel was tested. As shown in Fig. S5, the 3 groups of hydrogels showed certain adsorption capacity for BCA protein within 48 h, among which PEGDA5/AM15/CLC hydrogels had the strongest adsorption capacity for BCA, reaching 24.56 ± 1.05 mg/g at 48 h. This is because the positively charged CHW enhances the adsorption effect of hydrogel on protein (negative charge), and after ultrasound, the arrangement of CHW is regular, so that PEGDA5/AM15/CLC hydrogel has the largest adsorption amount of BCA protein. At the same time, PEGDA5/AM15/CLC hydrogel also showed excellent in vitro mineralization performance, and the calcium–phosphorus ratio of mineralized hydroxyapatite could reach 1.58 (Fig. S6), which was close to human bone tissue [39]. These excellent and in vitro mineralization abilities can better promote the adhesion, proliferation, and differentiation of cells on the scaffold material, so as to promote faster tissue repair [39].

Because of its huge specific surface area, variable size/pore structure, good biological activity, and highly organized mesoporous structure, MBG has drawn a lot of attention in the field of bone regeneration [33,40]. TEM (Fig. 5D) and SEM (Fig. 5E) images show that the prepared MBG scaffolds have uniform and ordered mesoporous channel structure and high specific surface area. The mesoporous pore size of the MBG is distributed in the range of 2 to 4 nm (Fig. 5F), and the N_2_ adsorption–desorption analysis for the MBG exhibits a typical IV isotherm pattern (Fig. 5G). Then, the degradation of the MBG in different pH solution environments was investigated (the degradation of the MBG was evaluated by the content of Ca^2+^ released after MBG degradation). The results (Fig. 5H and I) showed that in acidic environment (pH 4.0), the MBG gradually degraded, and more Ca^2+^ were released. In neutral and alkaline environments (pH 7.4 and pH 9.0), the MBG degradation was relatively slow, and the cumulative release of Ca^2+^ at 8 h was less than 1 mg, less than one-third of the release of calcium ions in acidic environments. Moreover, MBG maintained a slow degradation rate in PBS solution (pH 7.4) for a long time, and the release of Ca^2+^ and Si^4+^ basically reached the equilibrium at 20 d, which were 235.98 ± 2.48 ppm and 58.84 ± 1.67 ppm, respectively (Fig. S7). Along with the degradation of MBG, the loaded anti-inflammatory and other drugs are gradually released, and the self-degradation of MBG releases inorganic ions (Ca^2+^ and Si^4+^) to further promote bone repair [41,42]. Therefore, the MBG and its related tissue engineering materials can automatically adjust the immune microenvironment of wound tissue in response to changes in the tissue environment during tissue repair, achieving rapid and accurate therapeutic effects.

### Evaluation of cytocompatibility of hydrogels

**Fig. 6.**
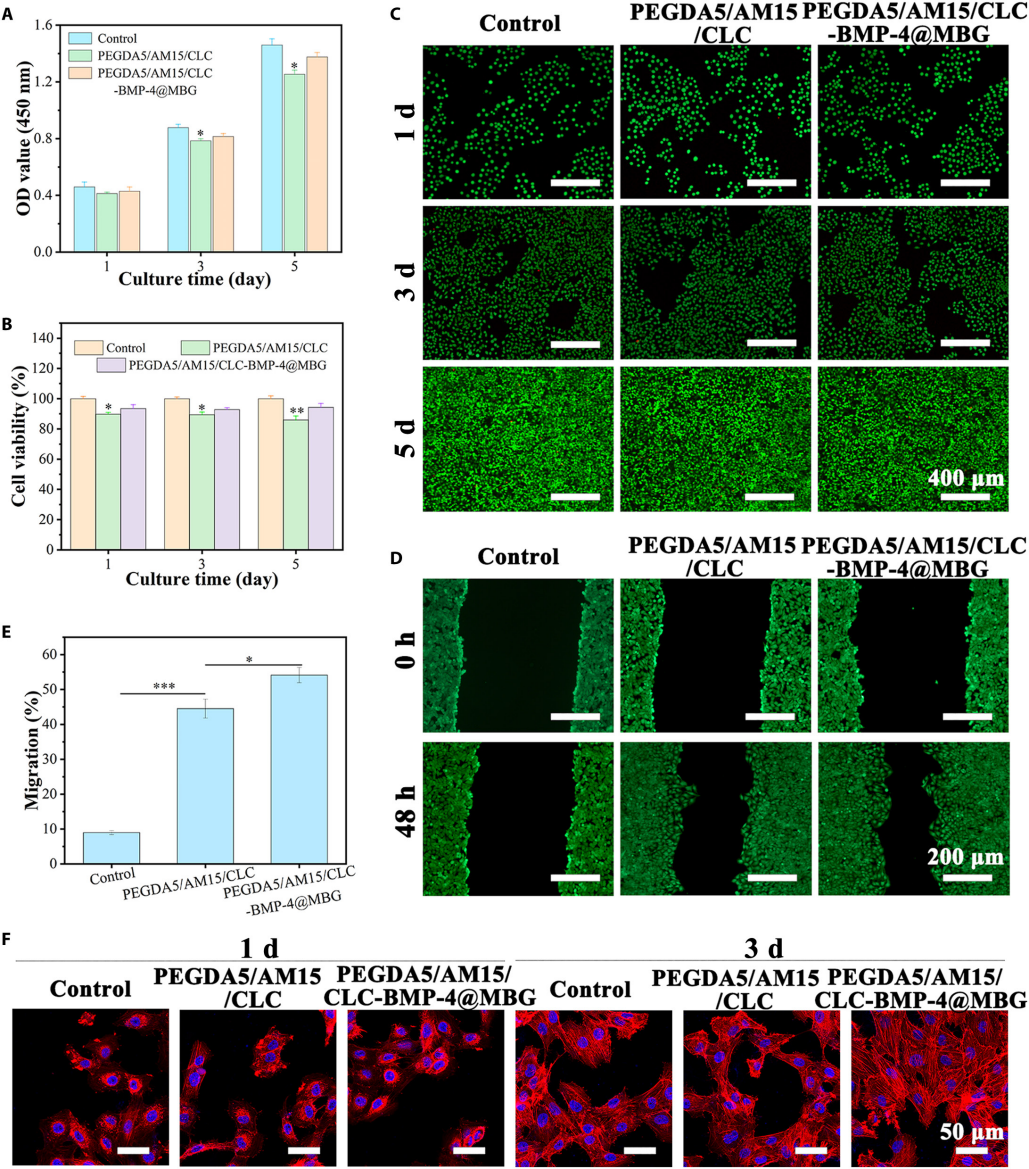
Evaluation of cell compatibility and cell migration promotion of hydrogels. (A) OD value, (B) cell viability, and (C) live and dead staining pictures of the cell coculture with hydrogel at 1, 3, and 5 d. (D and E) The migration results of MC3T3-E1 in coculture with culture plate and hydrogels. (F) CLSM images.

In the biomedical field, assessing the biocompatibility of biomedical materials is of utmost importance. To investigate the proliferation of MC3T3-E1 cells cocultured with hydrogels, the Cell Counting Kit-8 assay was employed. This assay is widely utilized to evaluate cell viability and proliferation and has proven to be a valuable tool in assessing the biocompatibility of materials in various biomedical applications. As shown in Fig. 6A, there was a noticeable increase in OD450 as the culture time increased for all 3 groups, indicating consistent cell proliferation. Additionally, the cell viability remained above 90% after coculture with the hydrogel for 1, 3, and 5 d, as compared to the control culture plates (Fig. 6B). This observation highlights the excellent cytocompatibility of the hydrogel material. Moreover, the loaded BMP-4 also actively promoted the proliferation of cells. Subsequently, the viability of MC3T3-E1 cells cocultured with hydrogels for different time periods was assessed using live/dead staining. According to the results presented in Fig. 6C, no significant differences were observed between the hydrogel-cocultured cells and the control group. Additionally, it was observed that as the culture time increased, the density of living cells significantly increased, while the number of dead cells decreased. These findings indicate that the hydrogel used in the coculture system supports cell proliferation and viability, suggesting its potential biocompatibility and suitability for biomedical applications. Simultaneously, analysis of cell morphology (Fig. 6F) revealed that with the increase of culture time, the spreading area of MC3T3-E1 cell coculture with the hydrogel with added BMP-4@MBG was larger compared to the other groups. Additionally, the cell displayed a distinctive elongated, spindle-shaped morphology with well-defined pseudopodia. These findings confirm the excellent cytocompatibility of the PEGDA5/AM15/CLC BMP-4@MBG hydrogel, indicating its potential suitability for various biological applications in the future, such as tissue engineering and regeneration.

Liquid crystal hydrogels have certain effects on cell migration due to their unique physical and chemical properties. Based on the results presented in Fig. 6D and E, it was observed that the PEGDA5/AM15/CLC and PEGDA5/AM15/CLC-BMP-4@MBG hydrogels significantly promoted cell migration in comparison to the blank control group. Specifically, after 48 h of culture with these hydrogels, the cell migration rates reached 44% and 54%, respectively. These findings suggest that the incorporation of CHWs in the hydrogels may have played a role in promoting cell migration and biological reconstruction. CHWs are known to be small natural particles that can potentially enhance cell adhesion, migration, and proliferation, making them a promising component in the design of biomaterials for tissue engineering applications. They can trigger signaling pathways by binding to integrin receptors on the cell surface, promoting the cell’s ability to adhere and migrate. Moreover, compared with the Control group and the PEGDA5/AM15/CLC group, PEGDA5/AM15/CLC-BMP-4@MBG also significantly promoted tubular formation of human umbilical vein endothelial cells (Fig. S8). This is mainly due to the combined action of BMP-4 and silicon ions released by MBG degradation [43–45]. This scaffold material with good cytocompatibility and vascularization ability will greatly improve its repair ability for bone tissue.

### Osteogenic properties of hydrogels

**Fig. 7.**
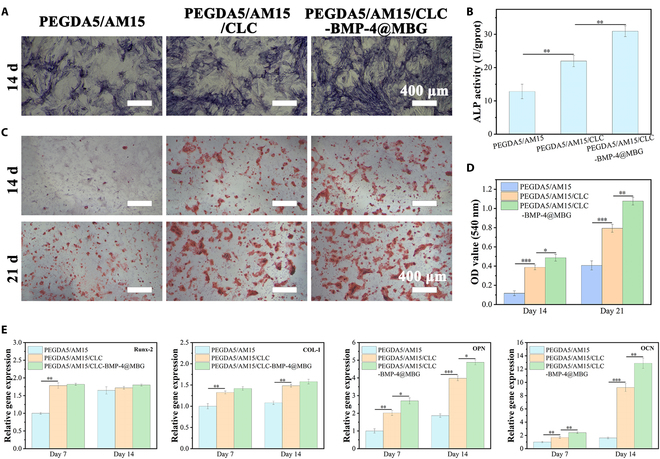
Evaluation of hydrogels promoting osteogenic differentiation. (A) ALP staining analysis and (B) quantitative analysis. (C) The Alizarin red staining analysis and (D) quantitative analysis. (E) Runx-2, COL-I, OPN, and OCN osteogenic gene expression levels following 7 and 14 d of cell growth on hydrogels.

ALP plays a crucial role as the primary indicator of early osteogenic cell development. In this study, we quantitatively and qualitatively analyzed the presence of ALP in cells that were cultivated on the hydrogel surface for a duration of 14 d. The objective was to investigate the impact of hydrogel samples on the early osteogenic differentiation of cells. The qualitative staining analysis results are presented in Fig. 7A. After a 14-d period of cell culture, it was observed that the liquid crystal hydrogels loaded with BMP-4@MBG exhibited significantly higher ALP activity compared to the other components. In the PEGDA5/AM15, PEGDA5/AM15/CLC, and PEGDA5/AM15/CLC-BMP-4@MBG groups, black-purple sediments increased progressively (Fig. 7B). The results suggest that the incorporation of CHWs and the subsequent formation of a liquid crystal structure, which resulted from the self-assembly of the whiskers, had a positive impact on the ALP activity of the cells. In addition, the liquid crystal hydrogel, when supplemented with BMP-4@MBG, created an even more favorable environment for early osteogenic differentiation of the cells.

On the other hand, the formation of calcium nodules serves as an indication of cell maturation and late-stage osteogenic differentiation. To evaluate the impact of the hydrogel on late osteogenic differentiation, Alizarin red staining was employed on cells cultured on the hydrogel for 14 and 21 d. The results, depicted in Fig. 7C and D, reveal a significantly higher number of red nodules in the hydrogel group compared to the PEGDA5/AM15 group, especially with a distinct accumulation of red nodules observed on the liquid crystal hydrogel of PEGDA5/AM15/CLC-BMP-4@MBG. Additionally, quantitative analysis provides a clearer distinction between the groups. The PEGDA5/AM15/CLC-BMP-4@MBG liquid crystal hydrogel exhibits a higher OD value, indicating its exceptional ability to induce osteogenic differentiation in comparison to the PEGDA5/AM15/CLC liquid crystal hydrogel without BMP-4@MBG.

Genes unique to osteoblasts are frequently expressed in conjunction with the process of cells differentiating into osteoblasts. Thus, in order to assess the osteoblastic activity of the cells on the hydrogel, we used reverse transcription PCR to assess the expression of osteoblastic genes. Figure 7E presents the test findings. Transcription factors Runx-2 and COL-I are essential for early osteogenic differentiation, while OPN and OCN function as markers for middle and late osteogenic differentiation. In contrast to cells cultured for 7 d, a significant increase in the expression of OCN and OPN genes was observed in cells cultured for 14 d, indicating middle- and late-stage osteogenic differentiation. However, since COL-I and Runx-2 are predominantly expressed during the early stage of osteogenic differentiation, their expression remained relatively unchanged or even decreased as the cells progressed toward the middle and late stages of osteogenic differentiation. Furthermore, the gene expressions of OCN, OPN, and COL-I in the PEGDA5/AM15/CLC and PEGDA5/AM15/CLC-BMP-4@MBG hydrogels were higher than those in the PEGDA5/AM15 hydrogels, indicating that the addition of CHWs and BMP-4@MBG could enhance the osteogenic differentiation capacity of cells on the hydrogels. In particular, the slowly released BMP-4, Ca^2+^, and Si^4+^ in hydrogels play a significant role in promoting cell osteogenesis [33,46].

### Evaluation of macrophage polarization regulation

**Fig. 8.**
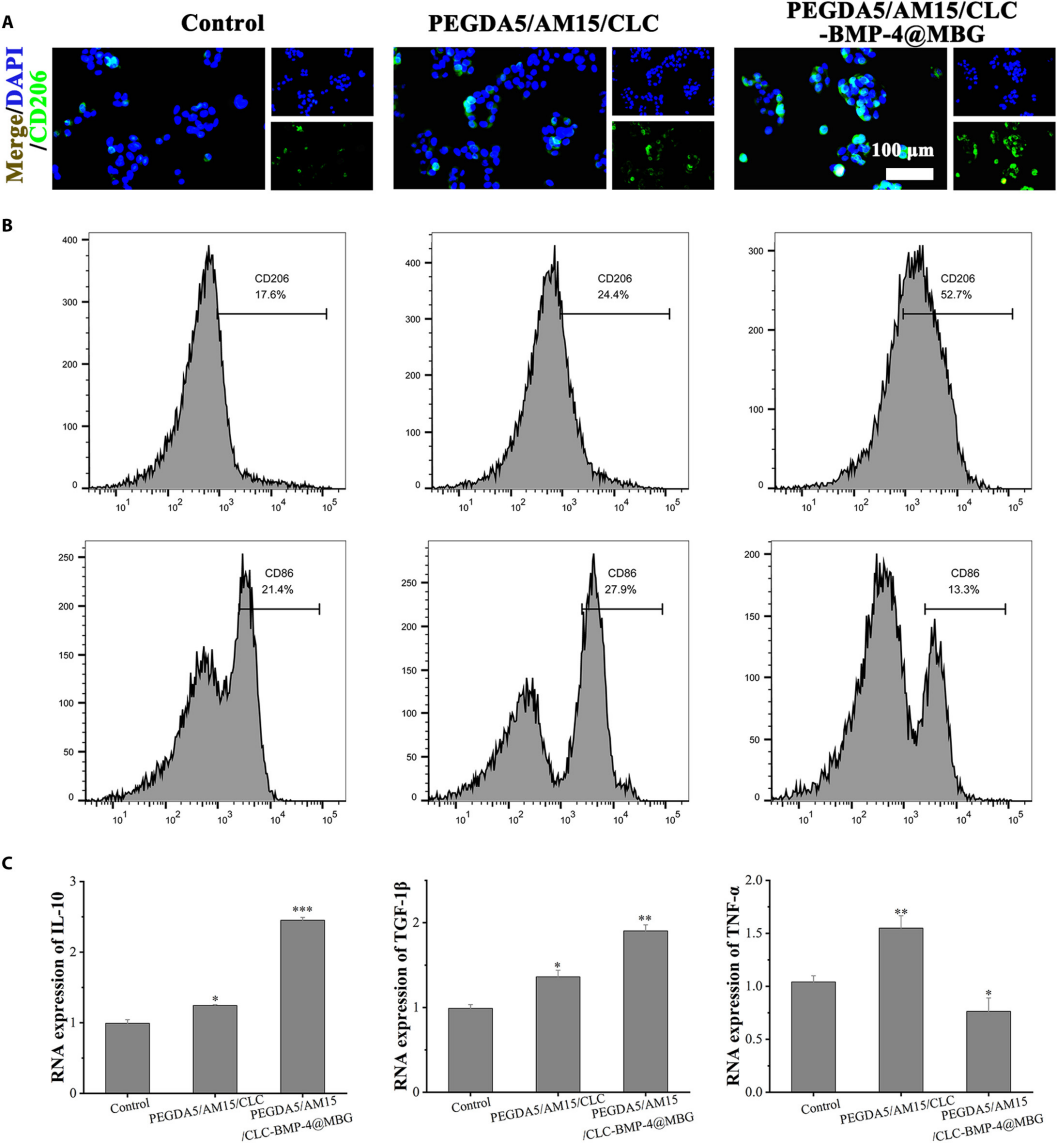
Evaluation of the effect of hydrogel on macrophage polarization. (A) Immunofluorescence staining of CD206 (green) in macrophages and (B) flow cytometry analysis of CD86^+^ macrophages and CD206^+^ macrophages treated with hydrogels for 48 h. (C) Macrophage-associated M1 or M2 marker gene expression (IL-10, TGF-1β, and TNF-α).

The process of skull injury repair is often accompanied by an inflammatory response that is difficult to control. M1 macrophages are present and produce a variety of large quantities proinflammatory cytokines exacerbate the inflammatory response while blocking it osteogenesis. Induced M2 polarization has been recognized as important for tissue repair, including bone regeneration. Therefore, the effect of hydrogel materials on the regulation of macrophage polarization toward the M1/M2 phenotype was further investigated. The CD206/DAPI immunofluorescence staining was used on macrophages to visualize the transformation of macrophages into M2 type. Immunofluorescence staining images (Fig. 8A) showed that CD206 expression was the most obvious in PEGDA5/AM15/CLC-BMP-4@MBG group, while only weak CD206 expression was found in the Control group. To evaluate macrophage polarization, CD206- and CD86-positive cells were identified and analyzed using flow cytometry. As illustrated in Fig. 8B, the highest proportion of M2 macrophages was exhibited by the PEGDA5/AM15/CLC-BMP-4@MBG group, with the lowest proportion of M1 macrophages. Conversely, the opposite pattern was displayed by the Control group. The RT-qPCR results (Fig. 8C) indicated an association of M2 macrophages with the PEGDA5/AM15/CLC-BMP-4@MBG group, wherein the anti-inflammatory cytokine IL-10 and the transforming growth factor TGF-1β were significantly expressed. Conversely, the proinflammatory cytokine TNF-α secreted by M1 macrophages was significantly down-regulated. This may be due to the liquid crystal microenvironment of the hydrogel and the release of BMP-4 to induce the transformation of macrophages to M2 type, which creates a favorable microenvironment for skull injury repair. BMP-4, by activating the Smad signaling pathway, not only directly induces osteogenic differentiation of BMSCs but also suppresses the production of proinflammatory cytokines (TNF-α) and promotes the transcription and expression of anti-inflammatory factors (IL-10) [46,47]. This process thereby facilitates the polarization of M1 macrophages to the M2 phenotype, mitigating the inflammatory response.

### Assessment of the ability to regenerate bone in vivo

**Fig. 9.**
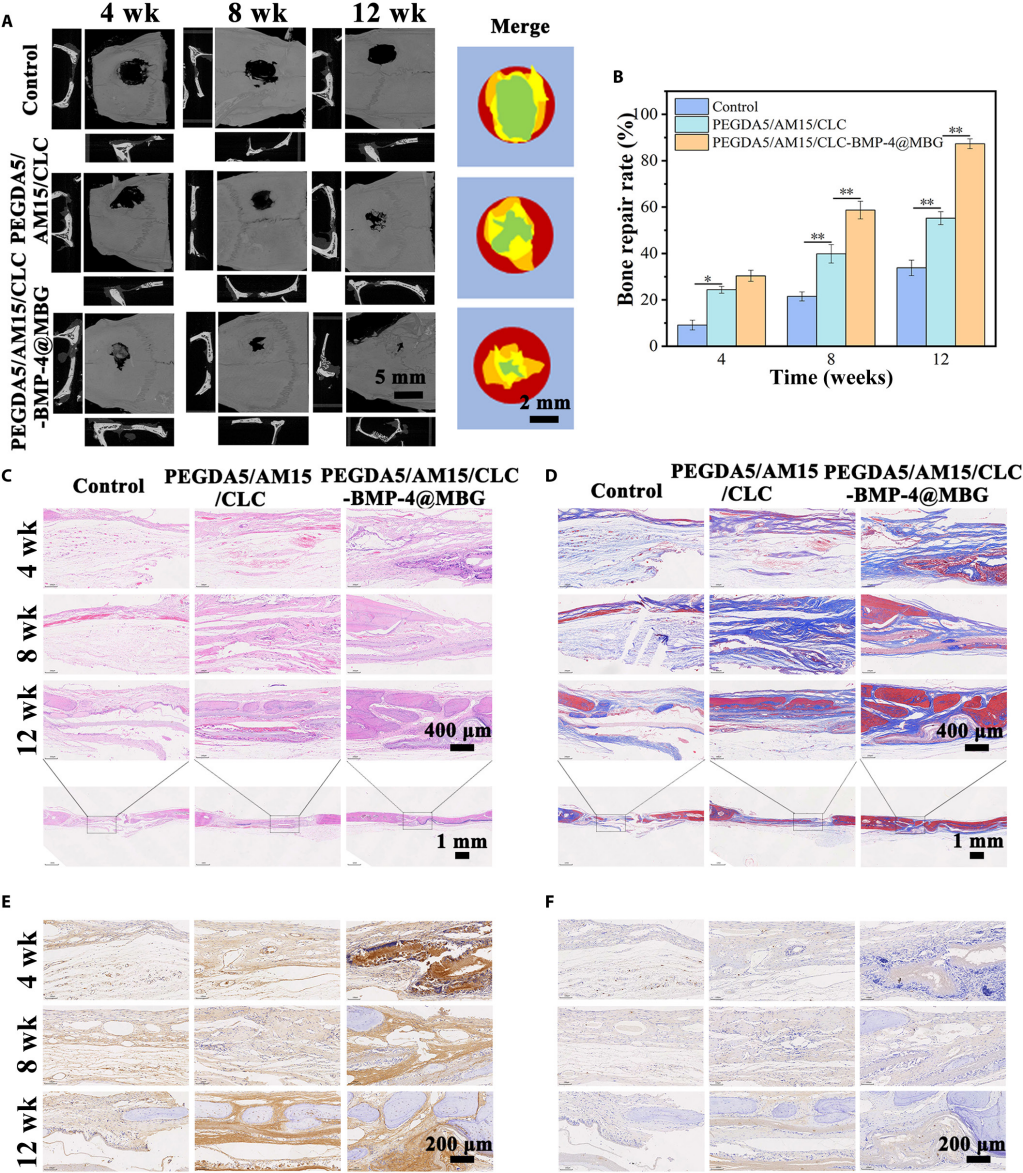
CT image and immunohistochemical analysis of skull tissue. (A) CT images of skull defect healing. (B) The rate of bone regeneration in different groups at 4, 8, and 12 weeks. (C) Stained rat skull repairs with H&E and (D) Masson in different groups after 4, 8, and 12 weeks. IHC staining for (E) OCN and (F) Runx-2 in different groups at 4, 8, and 12 weeks.

The hydrogel scaffold, PEGDA5/AM15/CLC-BMP-4@MBG, exhibits excellent physicochemical properties. To determine its repair ability for bone defects, we established a rat skull defect model and observed the repair process for 12 weeks. It could be intuitively seen from CT images (Fig. 9A) that with the increase of repair time, the damaged skulls in each group were continuously healing. The healing of the skull defect was not evident in the Control group, whereas in the PEGDA5/AM15/CLC-BMP-4@MBG group, the repair rate was importantly accelerated compared to both the PEGDA5/AM15/CLC group and the Control group. This is due to the liquid crystal hydrogel’s ability to provide a suitable microenvironment for bone cell proliferation and differentiation, while the slow release of MBG and BMP-4 promotes the synthesis and secretion of bone matrix, accelerating the repair and regeneration of bone tissue. The optical photos of the skull show the excellent progress of the PEGDA5/AM15/CLC-BMP-4@MBG hydrogel repair, with the skull defect being mostly healed after 12 weeks (Fig. 9B).

Furthermore, during the 12 weeks following material implantation, the development of new bone at the site of the skull defect was assessed via H&E staining (Fig. 9C). After 4 weeks postsurgery, osteoblasts were observed at the defect site in all 3 groups, with the PEGDA5/AM15/CLC-BMP-4@MBG group displaying a few new bone trabeculae. By the eighth week postsurgery, a higher expression of osteogenic markers was evident in the PEGDA5/AM15/CLC-BMP-4@MBG group compared to both the Control and PEGDA5/AM15/CLC groups. Moreover, a significant number of osteoblasts were observed to aggregate alongside a greater quantity of newly formed bone tissue. At the 12-week mark, the PEGDA5/AM15/CLC-BMP-4@MBG group displayed continuous new bone production at the skull defect, indicating effective bone healing in comparison to the Control and PEGDA5/AM15/CLC groups. Additionally, Masson’s staining examination (Fig. 9D) clearly depicts bone growth as collagen in the osteoid. The water-soluble dyes, aniline blue and xylidine Ponceau, can be utilized in the bone matrix to distinguish between old and new bone (the blue areas are new bone tissue and the red areas are mature bone tissue). After 4 weeks postoperation, collagen deposition at the bone defect site was notably higher in the PEGDA5/AM15/CLC-BMP-4@MBG group compared to the other 2 groups. At the 12th week after surgery, bone tissue in PEGDA5/AM15/CLC-BMP-4@MBG group was basically mature, and large bone tissue was also formed in PEGDA5/AM15/CLC group, while no continuous bone tissue was formed in the Control group. Chitin liquid crystal hydrogel could provide a liquid crystal state and viscoelastic environment similar to that of bone ECM, and has good bone promoting activity. In addition, the PEGDA5/AM15/CLC-BMP-4@MBG hydrogel intelligently responds to changes in bone microenvironment, releasing BMP-4, Ca^2+^, and Si^4+^ from MBG degradation, inhibiting the proliferation of inflammatory response and further promoting osteogenic differentiation, which more actively promote the bone tissue regeneration process.

To further validate the development of newly generated osseous tissues, immunohistochemical experiments were conducted with a specific focus on analyzing the expression levels of proteins associated with osteogenesis, such as Runx-2 and OCN. The Runx-2 staining analysis revealed that the PEGDA5/AM15/CLC-BMP-4@MBG group had significantly higher Runx-2 expression levels than the other 2 groups at various time intervals (Fig. 9F). Moreover, the expression of OCN exhibited similar outcomes (Fig. 9E). Additionally, the Runx-2 and OCN expression levels of the PEGDA5/AM15/CLC group were significantly higher than those of the Control group. These results suggest that the use of the MBG liquid crystal hydrogel scaffolds coated with BMP-4 can promote bone regeneration through microenvironmental immune regulation.

**Fig. 10.**
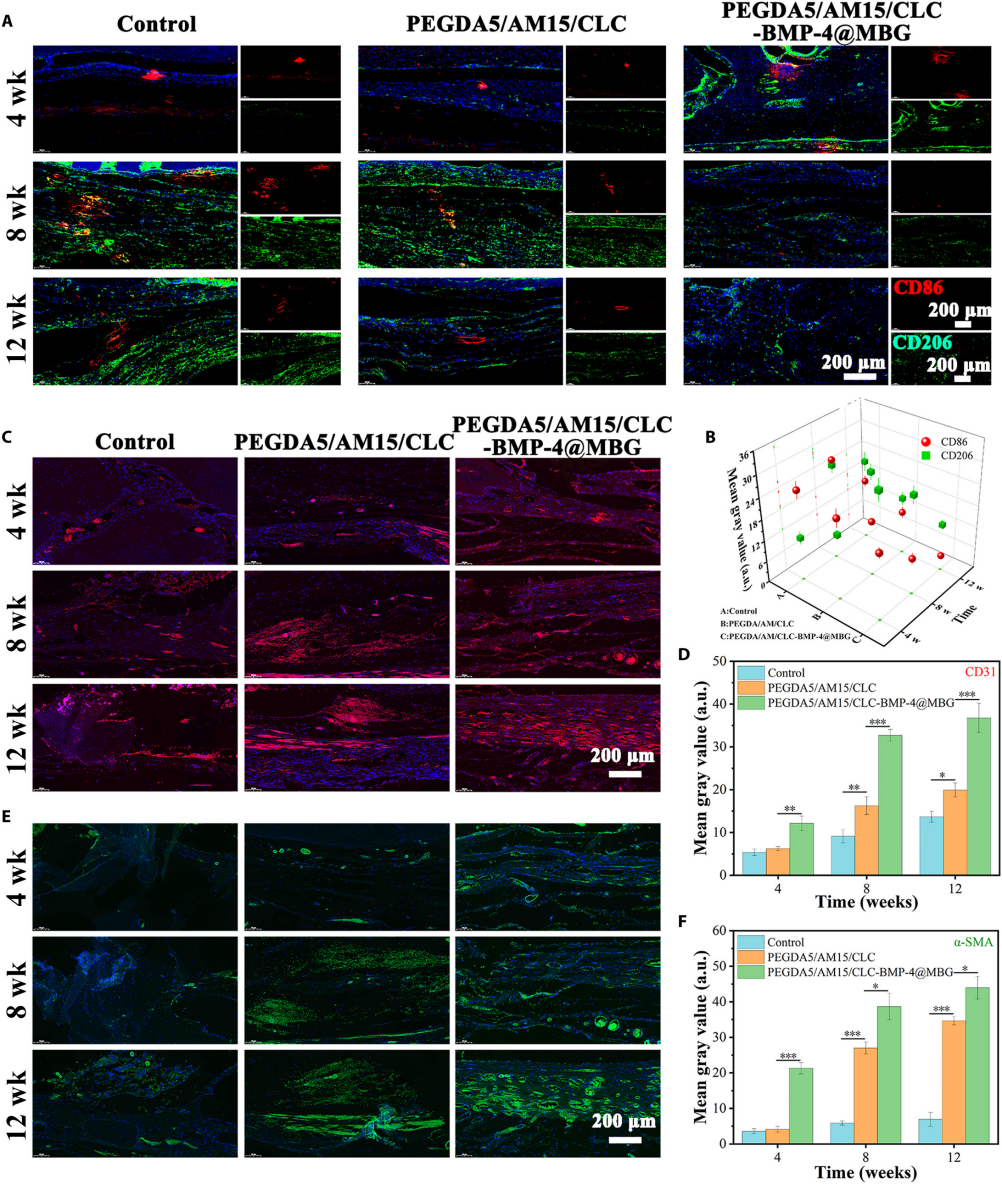
Analysis of inflammation and vascular remodeling in skull tissue. (A) Representative immunofluorescence images of CD86 (red, M1 phonotype), and CD206 (green, M2 phonotype) at 4, 8, and 12 weeks after surgery. (B) Fluorescence quantitative analysis of CD86 and CD206. (C) Fluorescence staining images and (D) fluorescence quantitative analysis of CD31. (E) Fluorescence staining images and (F) fluorescence quantitative analysis of α-SMA.

When macrophages shift from a proinflammatory phenotype (M1) to an anti-inflammatory phenotype (M2), the defective tissue moves from the inflammatory proliferative phase to the remodeling phase, a process closely linked to tissue regeneration progress. As shown in Fig. 10A and B, a large number of M1 macrophages (red, CD86-positive) were found in the tissue sections of each group at an early stage, indicating proinflammatory reaction. However, compared with other groups, the number of M2 macrophages (green, CD206-positive) in the PEGDA5/AM15/CLC-BMP-4@MBG group showed the highest, indicating that tissue repair was progressing from the proliferative stage to the remodeling stage. As time progressed, the fluorescence intensity of CD86 and CD206 in the PEGDA5/AM15/CLC and PEGDA5/AM15/CLC-BMP-4@MBG groups gradually diminished, suggesting the normal and systematic advancement of tissue repair. Conversely, the control group exhibited a more pronounced CD86 fluorescence, indicative of inflammatory cell presence. The PEGDA5/AM15/CLC-BMP-4@MBG scaffold material not only provides a suitable microenvironment for cell growth but also continuously releases BMP-4 to regulate the inflammatory microenvironment and promote the process of tissue repair quickly and effectively.

Blood vessel regeneration is essential for encouraging bone healing. Using CD31 and α-SMA immunofluorescence labeling, tissue samples were taken at 4, 8, and 12 weeks to evaluate the degree of vascularization during bone healing. Fluorescent staining images (Fig. 10C and E) showed that with the passage of repair time, more and more new blood vessels were found at the wound site, and the degree of vascularization in the Control group, PEGDA5/AM15/CLC group, and PEGDA5/AM15/CLC-BMP-4@MBG group increased successively. Semiquantitative fluorescence analysis of CD31 and α-SMA was performed using ImageJ, and the quantitative fluorescence results (Fig. 10D and F) agreed with the fluorescence images. To assess the biosafety and histocompatibility of the hydrogels, major organs (heart, liver, spleen, lungs, and kidneys) were collected from different treatment groups, and H&E staining was performed (Fig. S9). The findings demonstrated that there was no discernible difference in the shape of tissue slices in the hydrogel group when compared to the normal group. The results show that the hydrogel has good biocompatibility, no obvious biotoxicity, and the hydrogel does not cause obvious pathological damage to major organs, which proves the safety of its application in vivo. In summary, the smart hydrogel loaded with MBGs regulate the tissue immune microenvironment, enhancing both osteogenesis and vascularization abilities and actively promoting the bone tissue repair process.

## Conclusion

In this study, a liquid crystal hydrogel of chitin loaded with mesopore bioactive glasses was developed, which can enhance the repair effect of skull defect by regulating the immune microenvironment in bone tissue. In bone tissue engineering, liquid crystal scaffolds (especially chitin liquid crystal scaffolds) are favored by most researchers because of the similar structure to bone tissue and providing a suitable microenvironment for bone tissue regeneration [20,48]. However, the microenvironment provided by a single liquid crystal stent material is also prone to contamination due to wound deterioration [49]. In this design scheme, BMP-4-loaded mesoporous bioactive is introduced into chitin liquid crystal hydrogel, so that it can respond to changes in wound environment, degrade and release BMP-4, Ca^2+^, and Si^4+^ to regulate inflammatory environment and osteogenic environment. Specifically, when severe inflammation occurs in wound tissue, BMP-4 will accelerate the release along with the collapse of MBG, promoting the transformation of macrophages into M2 type, and making the repair process proceed in an orderly manner. Subsequently, Ca^2+^ and Si^4+^ released by MBG degradation can actively promote cell osteoblast differentiation and accelerate the process of bone repair. This kind of liquid crystal smart hydrogel has great application potential in the field of bone tissue regeneration due to its excellent biocompatibility, bioactivity, and immunomodulatory properties.

In conclusion, a chitin liquid crystal hydrogel loaded with MBGs for skull defect repair was developed inspired by natural bone ECM. A liquid crystal hydrogel was created using free radical polymerization of liquid crystal hydrogel precursor containing PEGDA and AM under UV irradiation. This hydrogel provides a viscoelastic and liquid crystal biotic environment and appropriate mechanical properties support and maintains the stability of CHW liquid crystal state. Additionally, it promoted osteoblast adhesion, proliferation, and differentiation, while the bioactivity of the liquid crystal hydrogel was further enhanced by the addition of BMP-4@MBG. The smart hydrogel can quickly respond to changes in the tissue microenvironment, release BMP-4, Ca^2+^, and Si^4+^, regulate the inflammatory environment of bone tissue and the component differentiation microenvironment, and thus promote the rapid and orderly process of tissue repair. As a result, notable improvements in skull defect restoration were observed. Both in vitro and in vivo experiments confirmed the remarkable cell affinity, inducing macrophages to M2 type transformation, osteogenesis, and angiogenesis capabilities of the PEGDA5/AM15/CLC-BMP-4@MBG liquid crystal hydrogel scaffold. With its viscoelasticity, exceptional mechanical stability, microenvironmental responsiveness, and bone-like ECM liquid crystal state, this liquid crystal hydrogel holds great promise as a material for bone healing.

## Data Availability

The materials and data obtained or analyzed in this study are available from the authors upon reasonable request.

## References

[1] Maruyama T, Jeong J, Sheu T-J, Hsu W. Stem cells of the suture mesenchyme in craniofacial bone development, repair and regeneration. Nat Commun. 2016;7(1):10526. 26830436 10.1038/ncomms10526 PMC4740445

[2] Mitra D, Whitehead J, Yasui OW, Leach JK. Bioreactor culture duration of engineered constructs influences bone formation by mesenchymal stem cells. Biomaterials. 2017;146:29–39. 28898756 10.1016/j.biomaterials.2017.08.044 PMC5618709

[3] Kwon H, Brown WE, Lee CA, Wang D, Paschos N, Hu JC, Athanasiou KA. Surgical and tissue engineering strategies for articular cartilage and meniscus repair. Nat Rev Rheumatol. 2019;15(9):550–570. 31296933 10.1038/s41584-019-0255-1 PMC7192556

[4] McDermott AM, Herberg S, Mason DE, Collins JM, Pearson HB, Dawahare JH, Tang R, Patwa AN, Grinstaff MW, Kelly DJ. Recapitulating bone development through engineered mesenchymal condensations and mechanical cues for tissue regeneration. Sci Transl Med. 2019;11(495):eaav7756. 31167930 10.1126/scitranslmed.aav7756 PMC6959418

[5] Liu H, Hu X, Li W, Zhu M, Tian J, Li L, Luo B, Zhou C, Lu L. A highly-stretchable and adhesive hydrogel for noninvasive joint wound closure driven by hydrogen bonds. Chem Eng J. 2023;452:139368.

[6] Liu Y, Hsu S-h. Synthesis and biomedical applications of self-healing hydrogels. Front Chem. 2018;6:449. 30333970 10.3389/fchem.2018.00449 PMC6176467

[7] Lee C-S, Singh RK, Hwang HS, Lee N-H, Kurian AG, Lee J-H, Kim HS, Lee M, Kim H-W. Materials-based nanotherapeutics for injured and diseased bone. Prog Mater Sci. 2023;135:101087.

[8] Zhang X, He Y, Huang P, Jiang G, Zhang M, Yu F, Zhang W, Fu G, Wang Y, Li W, et al. A novel mineralized high strength hydrogel for enhancing cell adhesion and promoting skull bone regeneration in situ. Compos Part B. 2020;197:108183.

[9] Zhu W, Chu C, Kuddannaya S, Yuan Y, Walczak P, Singh A, Song X, Bulte JW. In vivo imaging of composite hydrogel scaffold degradation using CEST MRI and two-color NIR imaging. Adv Funct Mater. 2019;29(36):1903753. 32190034 10.1002/adfm.201903753 PMC7079757

[10] Li D, Zhang K, Shi C, Liu L, Yan G, Liu C, Zhou Y, Hu Y, Sun H, Yang B. Small molecules modified biomimetic gelatin/hydroxyapatite nanofibers constructing an ideal osteogenic microenvironment with significantly enhanced cranial bone formation. Int J Nanomedicine. 2018;7167–7181. 30464466 10.2147/IJN.S174553 PMC6228053

[11] Sun X, Kang Y, Bao J, Zhang Y, Yang Y, Zhou X. Modeling vascularized bone regeneration within a porous biodegradable CaP scaffold loaded with growth factors. Biomaterials. 2013;34(21):4971–4981. 23566802 10.1016/j.biomaterials.2013.03.015 PMC3770300

[12] Yang Q, Han Y, Liu P, Huang Y, Li X, Li W. Long noncoding RNA GAS5 promotes osteogenic differentiation of human periodontal ligament stem cells by regulating GDF5 and p38/JNK signaling pathway. Front Pharmacol. 2020;11:518895. 10.3389/fphar.2020.00701 PMC7251029 32508644

[13] Huang Y-z, Ji Y-r, Kang Z-w, Li F, Ge S-f, Yang D-P, Ruan J, Fan X-q. Integrating eggshell-derived CaCO3/MgO nanocomposites and chitosan into a biomimetic scaffold for bone regeneration. Chem Eng J. 2020;395:125098.

[14] Kim H-S, Lee J-H, Mandakhbayar N, Jin G-Z, Kim S-J, Yoon J-Y, Jo SB, Park J-H, Singh RK, Jang J-H, et al. Therapeutic tissue regenerative nanohybrids self-assembled from bioactive inorganic core/chitosan shell nanounits. Biomaterials. 2021;274:120857. 33965799 10.1016/j.biomaterials.2021.120857

[15] Singh RK, Kurian AG, Patel KD, Mandakhbayar N, Lee N-H, Knowles JC, Lee J-H, Kim H-W. Label-free fluorescent mesoporous bioglass for drug delivery, optical triple-mode imaging, and photothermal/photodynamic synergistic cancer therapy. ACS Appl Bio Mater. 2020;3(4):2218–2229. 10.1021/acsabm.0c00050 35025274

[16] Guille MMG, Mosser G, Helary C, Eglin D. Bone matrix like assemblies of collagen: From liquid crystals to gels and biomimetic materials. Micron. 2005;36(7-8):602–608. 16169238 10.1016/j.micron.2005.07.005

[17] Lausch AJ, Chong LC, Uludag H, Sone ED. Multiphasic collagen scaffolds for engineered tissue interfaces. Adv Funct Mater. 2018;28(48):1804730.

[18] Bai X, Lü S, Liu H, Cao Z, Ning P, Wang Z, Gao C, Ni B, Ma D, Liu M. Polysaccharides based injectable hydrogel compositing bio-glass for cranial bone repair. Carbohydr Polym. 2017;175:557–564. 28917901 10.1016/j.carbpol.2017.08.020

[19] Hu H, Zhao Z, Sun Y, Zhao J, Chen Z, Li J, Kong W, Du Y, Shao J, Zhu X. 3D printing of porous degradable scaffolds with polylactic acid-loaded bioactive glass and calcium sulfate hemihydrate for bone defect repair. Adv Mater Technol. 2023;8(15):2300061.

[20] Chen J, Zhu Z, Chen J, Luo Y, Li L, Liu K, Ding S, Li H, Liu M, Zhou C, et al. Photocurable liquid crystal hydrogels with different chargeability and tunable viscoelasticity based on chitin whiskers. Carbohydr Polym. 2023;301(Part A):120299. 36436865 10.1016/j.carbpol.2022.120299

[21] Li L, Liu K, Chen J, Wen W, Li H, Li L, Ding S, Liu M, Zhou C, Luo B. Bone ECM-inspired biomineralization chitin whisker liquid crystal hydrogels for bone regeneration. Int J Biol Macromol. 2023;231:123335. 36690237 10.1016/j.ijbiomac.2023.123335

[22] Price JC, Roach P, El Haj AJ. Liquid crystalline ordered collagen substrates for applications in tissue engineering. ACS Biomater Sci Eng. 2016;2(4):625–633. 33465864 10.1021/acsbiomaterials.6b00030

[23] Zhang Z, Ma Z, Song L, Farag MA. Maximizing crustaceans (shrimp, crab, and lobster) by-products value for optimum valorization practices: A comparative review of their active ingredients, extraction, bioprocesses and applications. J Adv Res. 2023;57:59–76. 37931655 10.1016/j.jare.2023.11.002 PMC10918363

[24] Guo X, Wang J, Chen L, Wang Z, Zhang Y, Fang L. Liquefied chitin-derived super tough, sustainable, and anti-bacterial polyurethane elastomers. Chem Eng J. 2023;465:143074.

[25] Luo Y, Li Y, Liu K, Li L, Wen W, Ding S, Huang Y, Liu M, Zhou C, Luo B. Modulating of bouligand structure and chirality constructed bionically based on the self-assembly of chitin whiskers. Biomacromolecules. 2023;24(6):2942–2954. 37259538 10.1021/acs.biomac.3c00419

[26] Ngasotter S, Sampath L, Xavier KM. Nanochitin: An update review on advances in preparation methods and food applications. Carbohydr Polym. 2022;291:119627. 35698419 10.1016/j.carbpol.2022.119627

[27] Dhananasekaran S, Palanivel R, Pappu S. Adsorption of methylene blue, bromophenol blue, and coomassie brilliant blue by α-chitin nanoparticles. J Adv Res. 2016;7(1):113–124. 26843977 10.1016/j.jare.2015.03.003 PMC4703491

[28] Ma Y, Xu S, Yue P, Cao H, Zou Y, Wang L, Long H, Wu S, Ye Q. Synthesis and evaluation of water-soluble imidazolium salt chitin with broad-spectrum antimicrobial activity and excellent biocompatibility for infected wound healing. Carbohydr Polym. 2023;306:120575. 36746566 10.1016/j.carbpol.2023.120575

[29] Wang Y, Guo J, Zhou L, Ye C, Omenetto FG, Kaplan DL, Ling S. Design, fabrication, and function of silk-based nanomaterials. Adv Funct Mater. 2018;28(52):1805305. 32440262 10.1002/adfm.201805305 PMC7241600

[30] Kang MS, Lee N-H, Singh RK, Mandakhbayar N, Perez RA, Lee J-H, Kim H-W. Nanocements produced from mesoporous bioactive glass nanoparticles. Biomaterials. 2018;162:183–199. 29448144 10.1016/j.biomaterials.2018.02.005

[31] Mahapatra C, Singh RK, Kim J-J, Patel KD, Perez RA, Jang J-H, Kim H-W. Osteopromoting reservoir of stem cells: Bioactive mesoporous nanocarrier/collagen gel through slow-releasing FGF18 and the activated BMP signaling. ACS Appl Mater Interfaces. 2016;8(41):27573–27584. 27649064 10.1021/acsami.6b09769

[32] Kim T-H, Singh RK, Kang MS, Kim J-H, Kim H-W. Gene delivery nanocarriers of bioactive glass with unique potential to load BMP2 plasmid DNA and to internalize into mesenchymal stem cells for osteogenesis and bone regeneration. Nanoscale. 2016;8(15):8300–8311. 27035682 10.1039/c5nr07933k

[33] Liu Y, Zhang S, Zhang X, Ji L, Yu H, Wang J, Liu C. Porous PLGA/MBG scaffold enhanced bone regeneration through osteoimmunomodulation. Compos Part B. 2024;272:111202.

[34] Kim T-H, Singh RK, Kang MS, Kim J-H, Kim H-W. Inhibition of osteoclastogenesis through siRNA delivery with tunable mesoporous bioactive nanocarriers. Acta Biomater. 2016;29:352–364. 26432439 10.1016/j.actbio.2015.09.035

[35] Zheng A, Wang X, Xin X, Peng L, Su T, Cao L, Jiang X. Promoting lacunar bone regeneration with an injectable hydrogel adaptive to the microenvironment. Bioact Mater. 2023;21:403–421. 36185741 10.1016/j.bioactmat.2022.08.031 PMC9483602

[36] Peng Y, Hui H, Yuanfang L, Demin J, Alain D. Elastomer reinforced with regenerated chitin from alkaline/urea aqueous system. ACS Appl Mater Interfaces. 2017;9(31):26460–26467. 28719186 10.1021/acsami.7b08294

[37] Liu W, Liu K, Zhu L, Li W, Liu K, Wen W, Liu M, Li H, Zhou C, Luo B. Liquid crystalline and rheological properties of chitin whiskers with different chemical structures and chargeability. Int J Biol Macromol. 2020;157:24–35. 32335108 10.1016/j.ijbiomac.2020.04.158

[38] Li W, He X, Liu K, Wen W, Lu L, Liu M, Zhou C, Luo B. Creating ultrastrong and osteogenic chitin nanocomposite hydrogels via chitin whiskers with different surface chemistries. ACS Sustain Chem Eng. 2020;8(47):17487–17499.

[39] Xiao Q, Wang H, Wang L, Diao J, Zhao L, He G, Wang T, Jiang X. Interfacial modification of hydrogel composite membranes for protein adsorption with cavitands as nano molecular containers. Sep Purif Technol. 2024;339:126438.

[40] Zhou D, Yan X, Xiao L, Wang J, Wei J. Gold capped mesoporous bioactive glass guides bone regeneration via BMSCs recruitment and drug adaptive release. Chem Eng J. 2024;487:150546.

[41] Yao J, He Q, Zheng X, Shen S, Hui J, Fan D. An injectable hydrogel system with mild photothermal effects combined with ion release for osteosarcoma-related bone defect repair. Adv Funct Mater. 2024;34(30):2315217.

[42] Xue P, Chang Z, Chen H, Xi H, Tan X, He S, Qiao H, Jiang X, Liu X, Du B. Macrophage membrane (MMs) camouflaged near-infrared (NIR) responsive bone defect area targeting nanocarrier delivery system (BTNDS) for rapid repair: Promoting osteogenesis via phototherapy and modulating immunity. J Nanobiotechnol. 2024;22(1):87. 10.1186/s12951-024-02351-5 PMC10908146 38429776

[43] Chen J, Liao S, Kong Y, Xu B, Xuan J, Zhang Y. 3D-printed mesoporous bioactive glass scaffolds for enhancing bone repair via synergetic angiogenesis and osteogenesis. Mater Des. 2023;232:112089.

[44] Lee SS, Kim JH, Jeong J, Kim SHL, Koh RH, Kim I, Bae S, Lee H, Hwang NS. Sequential growth factor releasing double cryogel system for enhanced bone regeneration. Biomaterials. 2020;257:120223. 32736254 10.1016/j.biomaterials.2020.120223

[45] Wang X, Yuan Z, Shafiq M, Cai G, Lei Z, Lu Y, Guan X, Hashim R, El-Newehy M, Abdulhameed MM. Composite aerogel scaffolds containing flexible silica nanofiber and tricalcium phosphate enable skin regeneration. ACS Appl Mater Interfaces. 2024;16(20):25843–25855. 38717308 10.1021/acsami.4c03744

[46] Sun X, Ma Z, Zhao X, Jin W, Zhang C, Ma J, Qiang L, Wang W, Deng Q, Yang H, et al. Three-dimensional bioprinting of multicell-laden scaffolds containing bone morphogenic protein-4 for promoting M2 macrophage polarization and accelerating bone defect repair in diabetes mellitus. Bioact Mater. 2021;6(3):757–769. 33024897 10.1016/j.bioactmat.2020.08.030 PMC7522044

[47] Sheng N, Xing F, Zhang Q-Y, Tan J, Nie R, Huang K, Li H-X, Jiang Y-L, Tan B, Xiang Z, et al. A pleiotropic SIS-based hydrogel with immunomodulation via NLRP3 inflammasome inhibition for diabetic bone regeneration. Chem Eng J. 2024;480:147985.

[48] Li Y, Tang S, Luo Z, Liu K, Luo Y, Wen W, Ding S, Li L, Liu M, Zhou C, et al. Chitin whisker/chitosan liquid crystal hydrogel assisted scaffolds with bone-like ECM microenvironment for bone regeneration. Carbohydr Polym. 2024;332:121927. 38431420 10.1016/j.carbpol.2024.121927

[49] Bi Z, Cai Y, Shi X, Chen J, Li D, Zhang P, Liu J. Macrophage-mediated immunomodulation in biomaterial-assisted bone repair: Molecular insights and therapeutic prospects. Chem Eng J. 2024;150631.

