# Supplementary material for: Immunoregulation in Skull Defect Repair with a Smart Hydrogel Loaded with Mesoporous Bioactive Glasses
Source: Biomater Res. 2024 Sep 6;28:0074. doi: 10.34133/bmr.0074 (PMC11378080; doi:10.34133/bmr.0074)
Supplement: Supplementary 1 — Figs. S1 to S9 Table S1 [file bmr.0074.f1.docx]

Supplementary Materials

**Immunoregulation in skull defect repair with a smart hydrogel loaded with mesoporous bioactive glasses**

Shiguo Yuan^1,2*^, Boyuan Zheng^3*^, Kai Zheng^1,2*^, Zhiheng Lai^1,2*^, Zihang Chen^4^, Jing Zhao^5^, Shaoping Li^5^, Xiaofei Zheng^3♯^, Peng Wu^6♯^, Huajun Wang^3♯^

1 Department of Orthopaedic, Hainan Traditional Chinese Medicine Hospital, Hainan Medical University, Haikou, 571924, China

2 Department of Orthopaedic, Hainan Traditional Chinese Medicine Hospital, Guangzhou University of Chinese Medicine, Guangzhou, 510388, China

3 Department of Sports Medicine, The First Affiliated Hospital, Guangdong Provincial Key Laboratory of Speed Capability, The Guangzhou Key Laboratory of Precision Orthopedics and Regenerative Medicine, Jinan University, Guangzhou, 510630, China

4 Department of psychology, Li Ka Shing Faculty of Medicine, State Key Laboratory of Brain and Cognitive Sciences, The University of Hong Kong, Hong Kong SAR, 999077, China

5 State Key Laboratory of Quality Research in Chinese Medicine, Institute of Chinese Medical Sciences, Department of Pharmaceutical sciences, Faculty of Health Sciences, University of Macau, Macau SAR, 519000, China

6 Department of Orthopedics, Shanghai Tenth People's Hospital, Tongji University School of Medicine, Shanghai, 200072, China

* These authors contributed equally to this work and should be considered co-first authors.

# Corresponding author.

E-mail address: zhengxiaofei12@163.com (X. Zheng); wupeng03010814@163.com (P. Wu); whj323@126.com (H. Wang)

**Methods:**

***In Vitro* Degradation Experiment**

The dry gel was weighed (*m*0) and placed in PBS solution containing 100 u/mL lysozyme. The hydrogel samples were removed at 1, 3, 5, 7, 14 and 21 days, respectively, and freeze-dried and weighed as *m*t. The morphologies of degraded hydrogels at day 3 and 14 were characterized by SEM. The culture medium was changed every two days. Degradation ratio (DR) is calculated according to the following formula:

$$DR=\frac{m0-mt}{m0}\times100\%$$

**BCA Adsorption Experiment**

First, 50 mg of freeze-dried hydrogel was placed in 10 mL PBS solution containing BCA (50 mg/mL), and part of the stock solution was taken at 2, 4, 6, 8, 24 and 48 h, respectively, and the bacteria were filtered by 0.22 μm filter head, and then the filtrate was diluted to the detectable range of the kit. The 4 μL solution to be tested was mixed with the working solution of the 200 μL BCA kit, incubated at 60 ℃ for half an hour, and then the absorbance at 562 nm was measured with microplate reader, and the corresponding protein concentration was calculated using the BCA standard curve. Finally, the following formula was used to calculate the protein adsorption capacity (AC) of each group of materials.

$$AC(mg/g)=\frac{\left( C0-Ct \right)\times V}{w}$$

*V* is the volume of the extract (mL), *w* is the mass of the hydrogel (g), *C*0 is the initial protein concentration, and *C*t is the protein concentration after adsorption.

***In Vitro* Mineralization Experiment**

Firstly, solution A containing 250 mL 1 mM potassium dihydrogen phosphate and 62.5ug/mL polyaspartate was prepared, and then solution B was prepared with 100 mL 0.16 mmoL calcium chloride. The hydrogel sample is then placed in solution A and solution B is added drop by drop to solution A. Finally, the mineralized liquid was adjusted to neutral and mineralized at 37 ^o^C for 48 h. After freeze-drying, SEM was used to characterize the mineralization of various materials.

**Tube Formation Assay**

Matrix glue (10 μL/well) was equally applied to the 96-well plate, which was then incubated for 30 minutes at 37 °C. After that, HUVECs (6000/well) were added to the well plate and cultured for one to two hours. After discarding the supernatant, new serum-free media containing various components was added to continue the culture and track the development of tubules.

Table S1. Primers for osteogenic related genes

| Prier | Forward sequence (5’-3’) | Reverse sequence (3’-5’) |
| --- | --- | --- |
| OCN | AGGGCAATAAGGTAGTGAA | CGTAGATGCGTCTGTAGGC |
| COL-Ⅰ | CTTCACCTACAGCACCCTTGT | AAGGGAGCCACATCGATGAT |
| OPN | ACCATTCGGATGAGTCTGAT | TCAGTCCATAAGCCAAGCTA |
| Runx-2 | TGCCCAGTGAGTAACAGAAAGAC | CTCCTCCCTTCTCAACCTCTAA |
| β-actin | GCTTCTAGGCGGACTGTTAC | CCATGCCAATGTTGTCTCTT |

**Results:**


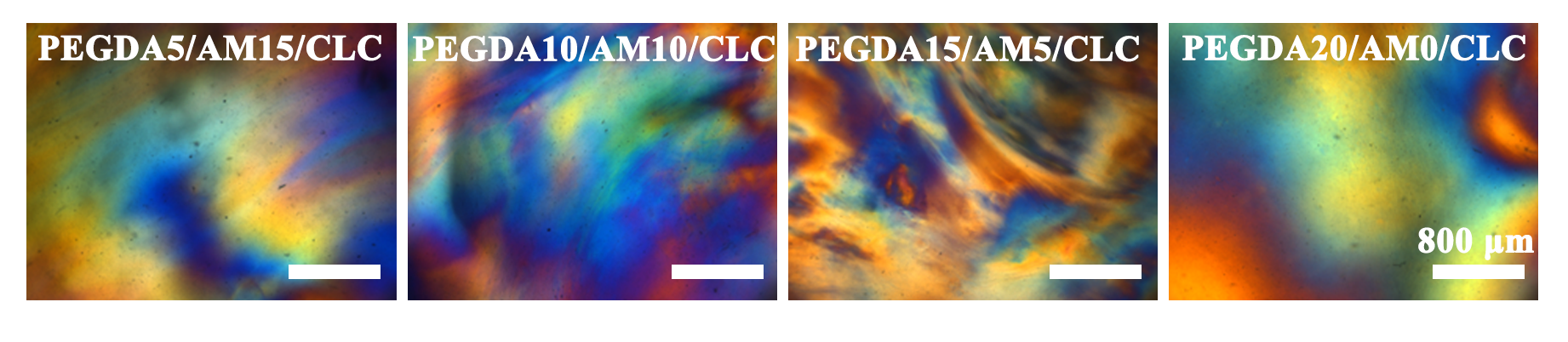


Fig. S1. Polarizing photos of liquid crystal hydrogels of different components.


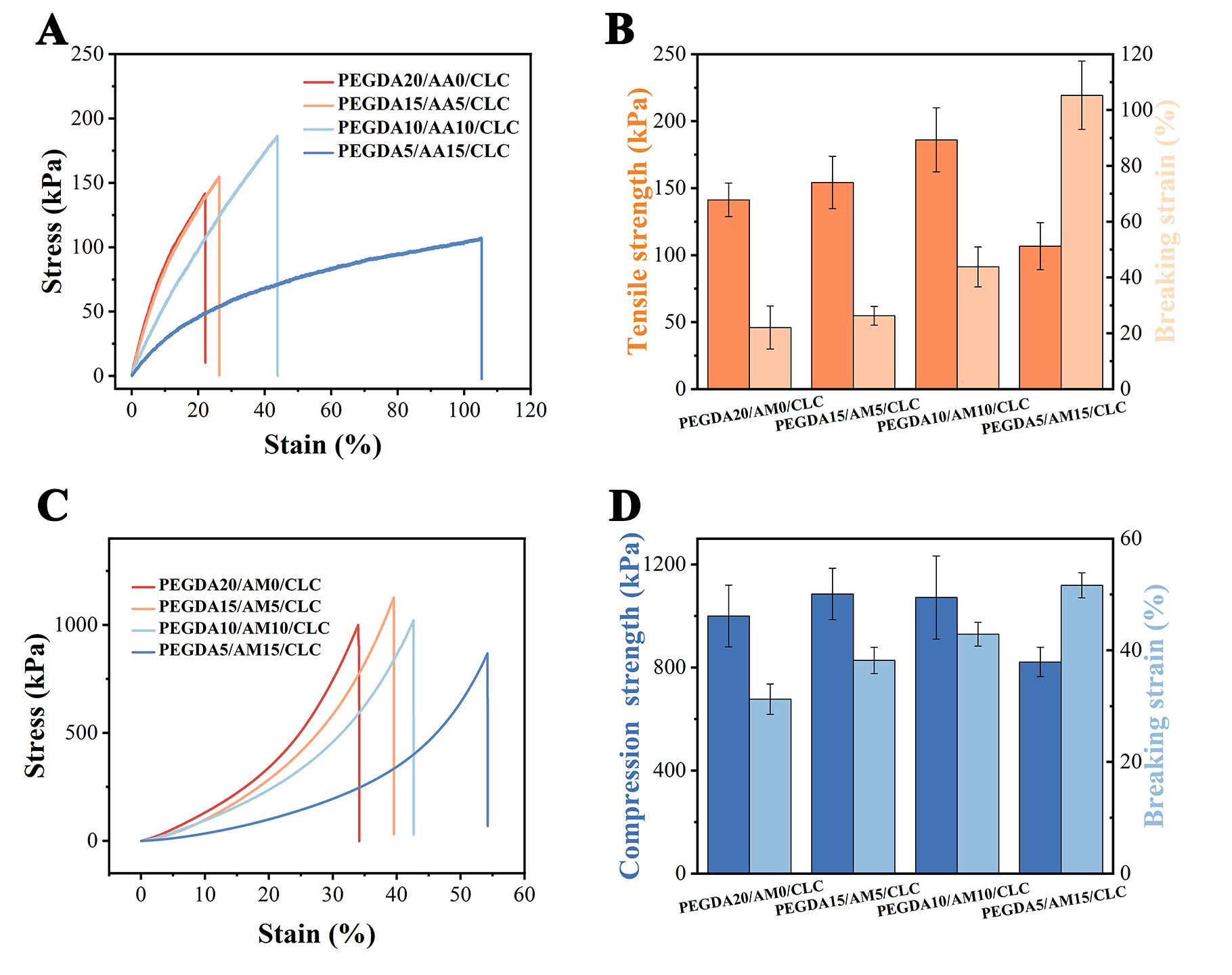


Fig. S2. (A) tensile curve, (B) tensile strength and fracture strain, (C) compressive curve, and (D) compressive strength and fracture strain of liquid crystal hydrogels.


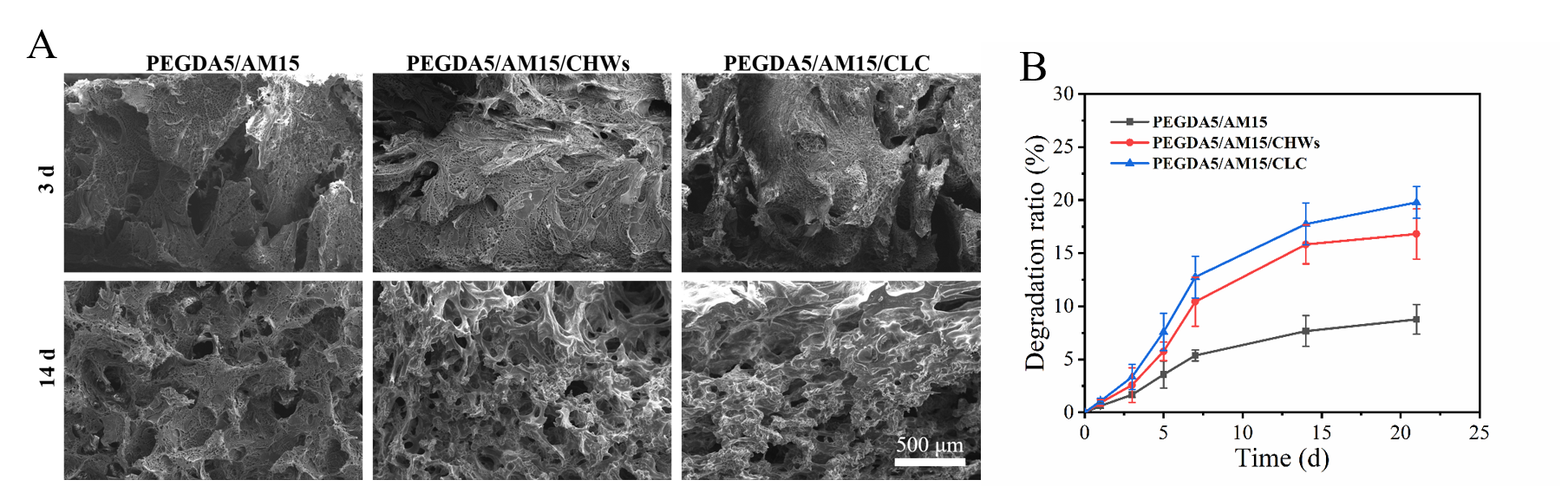


Fig. S3. (A) SEM images of hydrogel at degradation of 3 and 14 days. (B) Degradation ratio of hydrogel within 21 days.


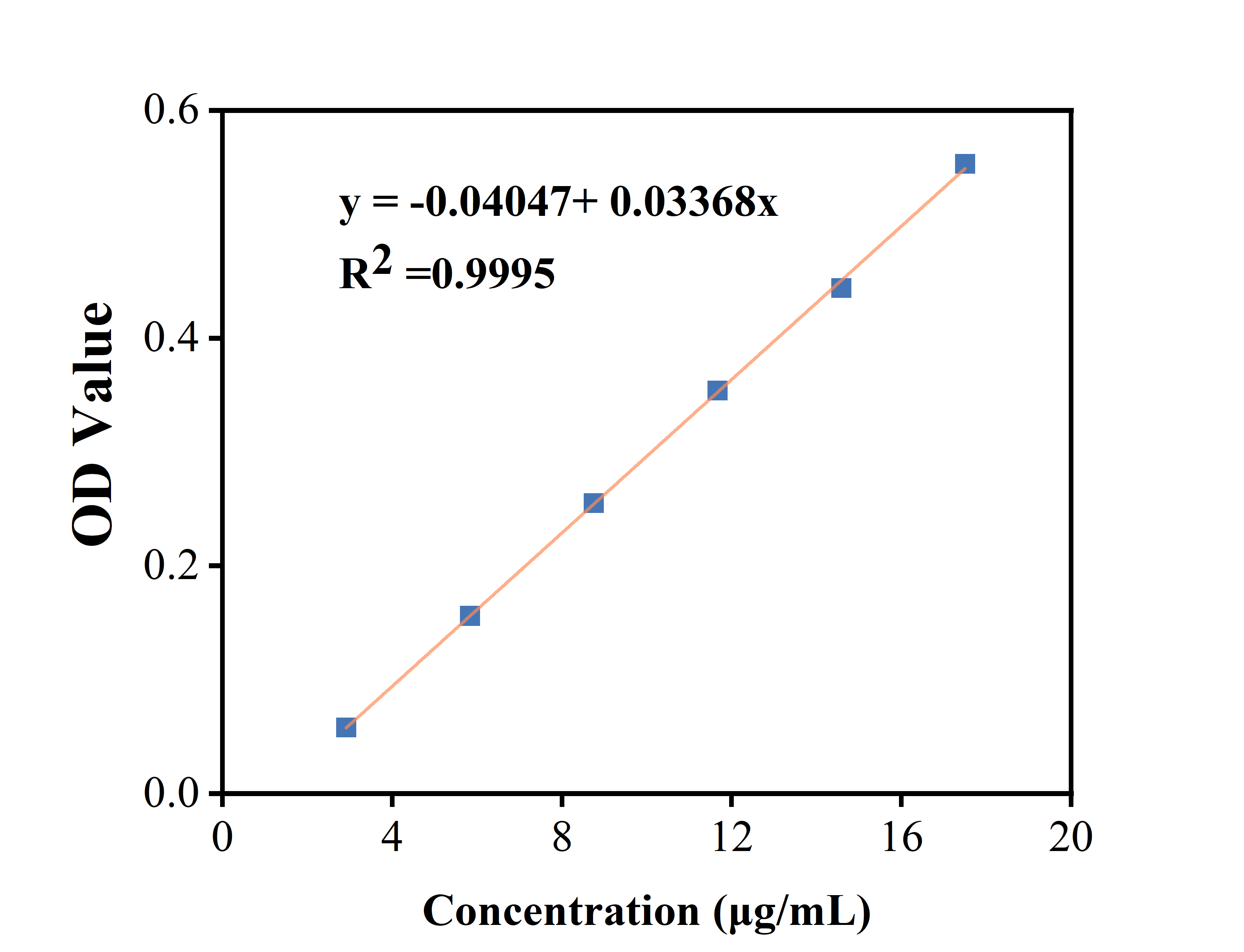


Fig. S4. The standard curve of Rh B aqueous solution





Fig. S5. The adsorption ratio of BCA protein by hydrogel within 48 h


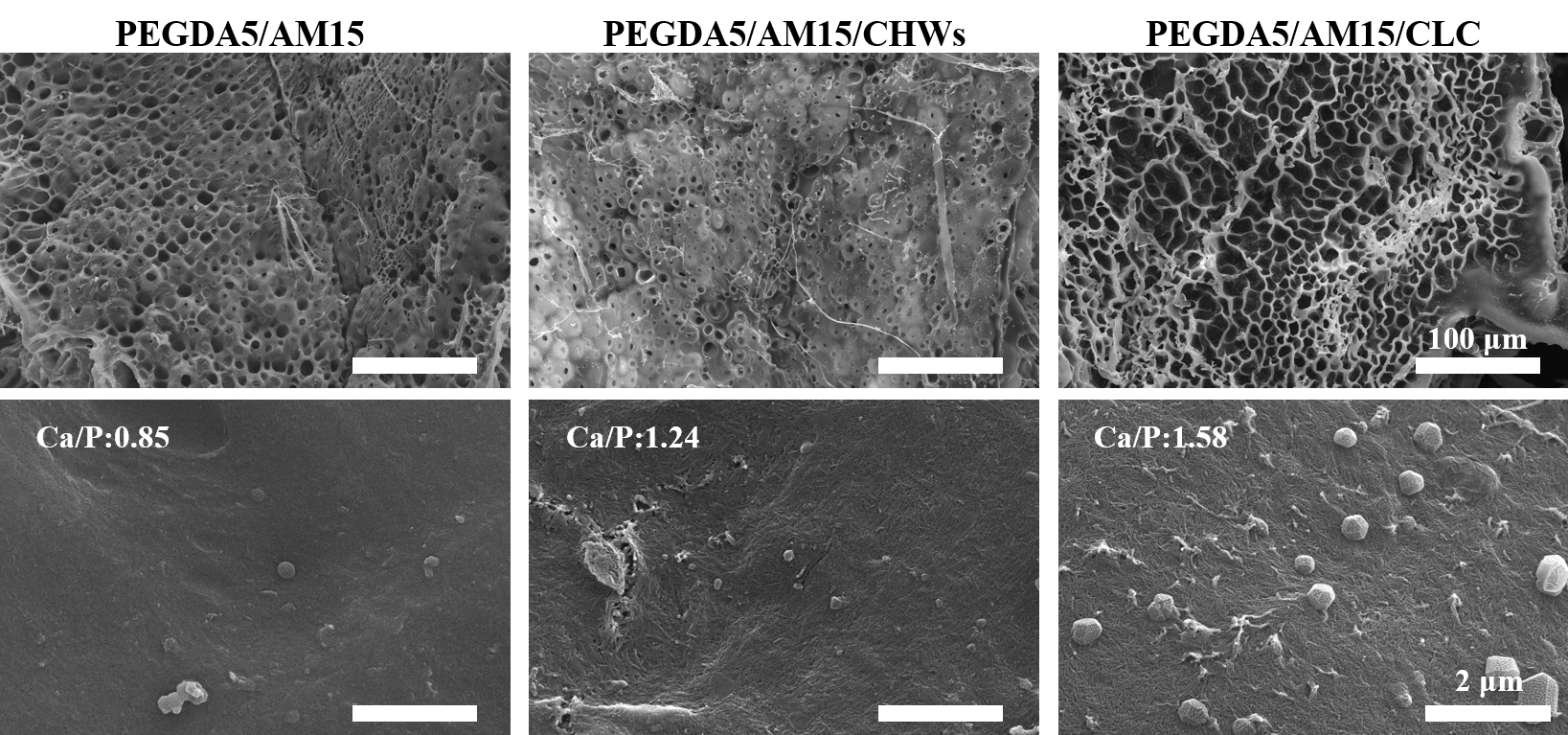


Fig. S6. SEM images of hydrogel after mineralization *in vitro*





Fig. S7. The cumulative release rate of Ca^2+^ and Si^4+^ in 20 days


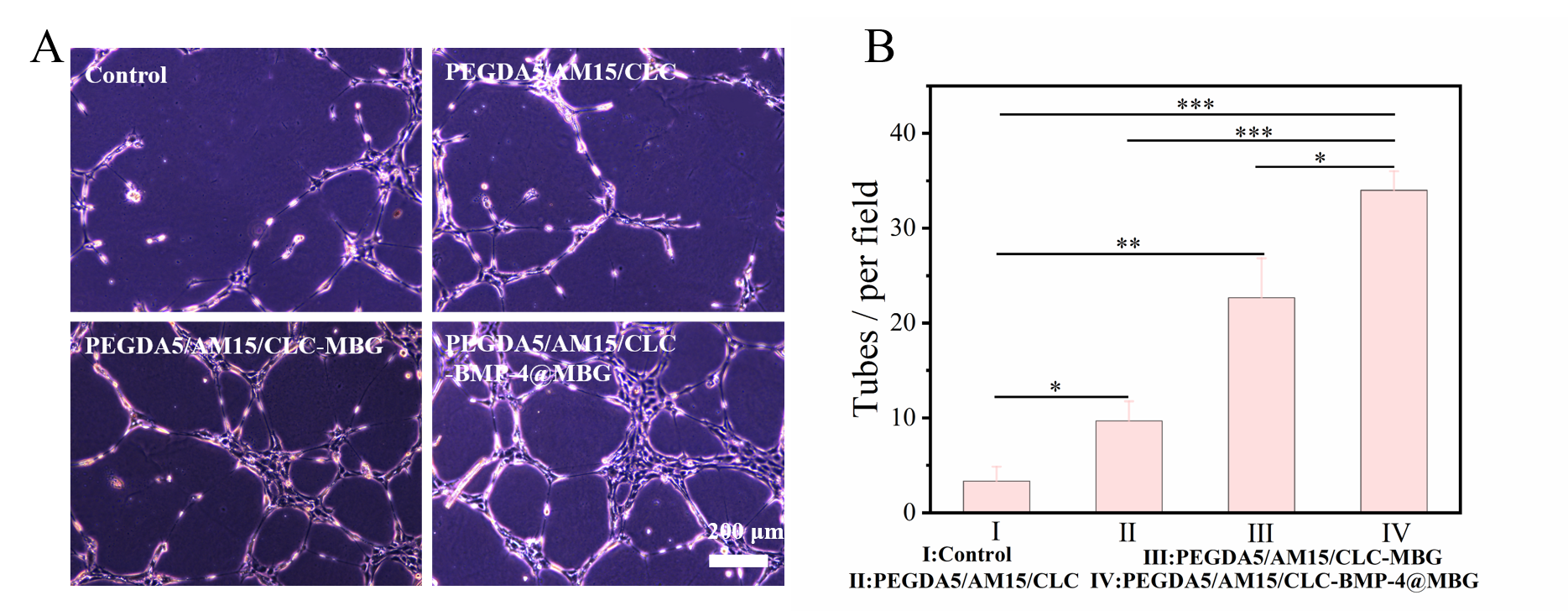


Fig. S8. (A) Photographs and (B) quantitative statistics of HUVECs tube formation *in vitro* with different groups


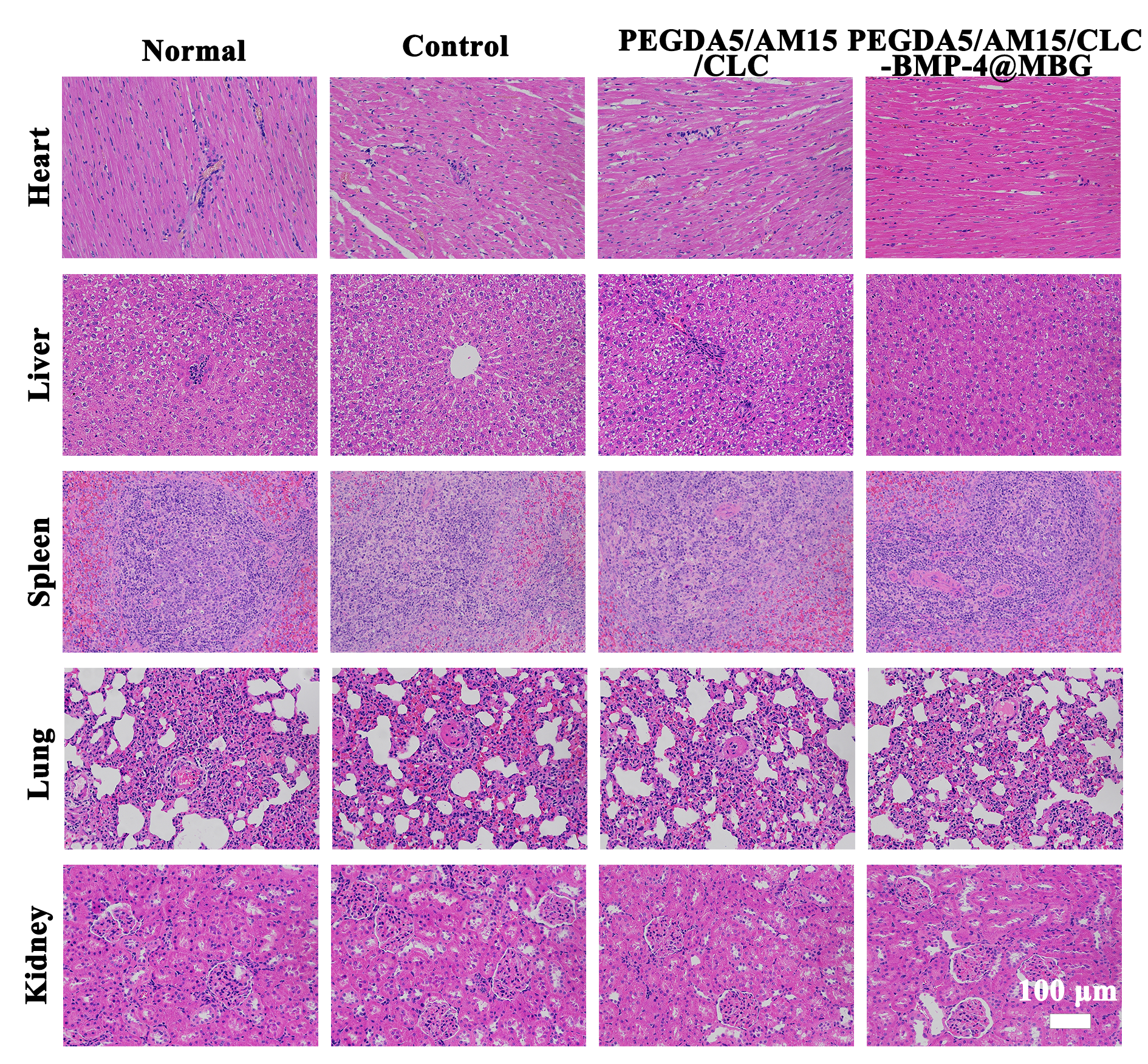


Fig. S9. HE staining of major organs (heart, liver, spleen, lungs and kidneys) in SD rats after 12 weeks of repair.
